# Niacin enhances hematoma clearance and neurological recovery via the HCAR2/SIRT1/Nrf2 pathway after germinal matrix hemorrhage

**DOI:** 10.1016/j.redox.2025.103916

**Published:** 2025-11-04

**Authors:** Xiong Liu, Cheng Cao, Ningbo Xu, Feng Xu, Lei Huang, Prativa Sherchan, Desislava Met Doycheva, John H. Zhang, Jiping Tang, Jerry J. Flores, Xionghui Chen

**Affiliations:** a Department of Emergency Surgery, First Affiliated Hospital of Soochow University, Suzhou, Jiangsu, 215000, China; b Department of Physiology and Pharmacology, Basic Sciences, School of Medicine, Loma Linda University, Loma Linda, CA, 92354, USA; c Department of Intensive Care Unit, The Affiliated Jiangyin Hospital of Nantong University, Jiangyin, Jiangsu, 214400, China; d Department of Brain Center, The Affiliated Jiangyin Hospital of Nantong University, Jiangyin, Jiangsu, 214400, China; e Department of Interventional Therapy, Zhujiang Hospital, Southern Medical University, Guangzhou, Guangdong, 510282, China; f Department of Neurosurgery, Loma Linda University School of Medicine, Loma Linda, CA, 92354, USA; g Department of Anesthesiology and Neurology, Loma Linda University School of Medicine, Loma Linda, CA, 92354, USA

**Keywords:** Germinal matrix hemorrhage (GMH), Hematoma, Neurological function, Niacin, HCAR2(hydroxycarboxylic acid receptor 2), Microglia

## Abstract

Germinal matrix hemorrhage (GMH) is a common form of neonatal stroke in preterm infants and often results in severe and lasting neurological deficits. Persistent hematoma is a key contributor to secondary brain injury. In this study, we investigated the therapeutic potential of niacin in promoting hematoma clearance and neuroprotection after GMH. We found that intranasal administration of niacin markedly improved neurological function, accelerated hematoma clearance, and attenuated secondary brain damage in neonatal rats. Mechanistically, niacin activated HCAR2(hydroxycarboxylic acid receptor 2) and further upregulated its expression, inducing microglial polarization toward the M2 phenotype through the SIRT1/Nrf2 pathway. This activation enhanced CD36/CD163/HO-1–mediated phagocytosis and degradation of erythrocytes and hemoglobin. Nuclear translocation of Nrf2 is essential for the effects of niacin. CRISPR-mediated knockout of HCAR2, or pharmacological inhibition of SIRT1 or Nrf2, abolished niacin-induced hematoma clearance and neuroprotection. Furthermore, microglial depletion significantly attenuated the protective effects of niacin. Collectively, niacin regulates microglial function via the HCAR2/SIRT1/Nrf2 signaling axis to reduce neuronal damage, neuroinflammation, and oxidative stress, thereby alleviating hydrocephalus and improving neurological outcomes. These findings suggest a potential non-invasive therapeutic strategy for neonatal GMH.

## Graphical abstract

**Figure:**
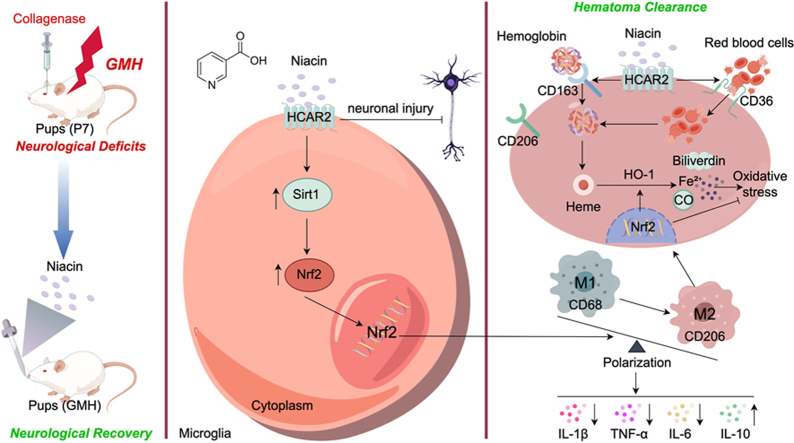

## 1 Introduction

Germinal matrix hemorrhage (GMH), a severe form of neonatal stroke, originates from the rupture of fragile vessels in the subependymal or periventricular germinal zone, often progressing to intraventricular hemorrhage (IVH), especially in preterm infants [1]. Despite advances in neonatal intensive care, GMH-IVH remains a major cause of neurological impairment—such as developmental delay, cerebral palsy, and cognitive deficits—in survivors, primarily due to posthemorrhagic hydrocephalus (PHH) [2,3]. Intraventricular clots obstruct arachnoid villi, disrupting cerebrospinal fluid dynamics and contributing to PHH [4]. Although cerebrospinal fluid (CSF) shunting remains the primary symptomatic treatment, it carries risks of obstruction and infection [5]. Thus, early non-invasive hematoma clearance before the onset of PHH symptoms is critical for preventing secondary brain injury.

The hematoma initiates a cascade of secondary destructive events. Lysed red blood cells (RBCs) release hemoglobin, which rapidly oxidizes and liberates the heme prosthetic group. Free heme is subsequently metabolized by heme oxygenase-1 (HO-1) into iron, carbon monoxide (CO), and biliverdin. These metabolites exert distinct biological effects: iron is particularly detrimental, as it promotes neuroinflammation, oxidative stress, and neuronal injury, whereas CO and biliverdin may confer protective effects under certain conditions [6,7]. Therefore, early clearance of endogenous hematoma during the initial phase of GMH pathophysiology is essential for mitigating secondary brain injury.

Microglia, the resident immune cells of the central nervous system (CNS), are rapidly activated and recruited to the hematoma site following hemorrhagic stroke [8]. Their phenotypic polarization plays a pivotal role in shaping the inflammatory milieu and influencing tissue outcome. Classically activated M1 microglia exacerbate neural injury by releasing proinflammatory mediators, whereas alternatively activated M2 microglia, which are enriched at the hemorrhagic core, facilitate hematoma clearance through enhanced phagocytosis, attenuate secondary injury, and promote neurological recovery [9,10]. Therefore, strategies that promote microglial polarization toward the M2 phenotype, along with mechanistic insights into endogenous regulatory pathways, may offer promising therapeutic avenues for clinical intervention in GMH.

HCAR2 (hydroxycarboxylic acid receptor 2) is a G protein–coupled receptor with high affinity for niacin [11]. In the central nervous system, HCAR2 is selectively expressed by microglia and senses various extracellular signals to regulate cellular activity [12]. In the brain, HCAR2 expression has been shown to regulate microglial activity in various central nervous system (CNS) diseases, offering potential for improving neuro pathology [13]. Specifically, activation of HCAR2 elicits a broad spectrum of microglia-mediated protective responses, leading to reduced infarct volume and attenuated brain injury in models of ischemic stroke [12]. Similarly, HCAR2 activation has been shown to alleviate amyloid burden, prevent neuronal loss, and improve neurological function in Alzheimer's disease [14]. An increasing number of studies have shown that the activation of Sirtuin 1 (SIRT1) is a key mechanism by which HCAR2 exerts its biological functions[[15], [16], [17]]. SIRT1 can deacetylate and activate Nrf2, thereby enhancing the transcription of antioxidant and cytoprotective genes [16,18,19]. In addition, niacin upregulates CD36 (cluster of differentiation 36) expression via HCAR2 activation, thereby enhancing microglial phagocytosis of myelin debris and exerting therapeutic effects in demyelination models [20]. Although accumulating evidence supports the neuroprotective effects of niacin in various neurological disorders—particularly in ischemic stroke—its role in hemorrhagic stroke, especially in germinal matrix hemorrhage (GMH), remains poorly understood.

In the present study, we initially showed that intranasal administration of niacin significantly improves neurological outcomes in neonatal rats after GMH, accelerates hematoma clearance, and attenuates secondary brain injury. Mechanistically, niacin upregulates HCAR2 expression and activates the HCAR2(hydroxycarboxylic acid receptor 2)/SIRT1(Silent Information Regulator 1)/Nrf2(Nuclear factor erythroid 2–related factor 2) signaling pathway, which drives microglial polarization toward the M2 phenotype. This shift enhances CD36(cluster of differentiation 36)/CD163(cluster of differentiation 163)/HO-1(heme oxygenase-1)–mediated phagocytosis and degradation of erythrocytes and hemoglobin, thereby reducing neuronal damage, neuroinflammation, and oxidative stress. Consequently, niacin attenuates hydrocephalus and alleviates neurological deficits.

## 2 Materials and methods

### 2.1 Animals

All experimental procedures were approved by the Institutional Animal Care and Use Committee (IACUC) of Loma Linda University (#23–030) and conducted in accordance with the National Institutes of Health (NIH) guidelines and the ARRIVE (Animal Research: Reporting of In Vivo Experiments) guidelines. A total of 422 postnatal day 7 (P7) Sprague-Dawley rat pups (weighing 12–14 g; Envigo, Indianapolis, IN), regardless of sex, were randomly assigned to either the Sham or GMH group. The developmental stage of the rat brain at P7 corresponds to that of a human infant at gestational age 30–32 weeks. Detailed experimental design (Supplementary Fig. S1) and group sample sizes (Supplementary Table S1) are provided in the Supplementary Information.

### 2.2 GMH model

As previously described [21], Grade III or IV germinal matrix hemorrhage (GMH) was induced by stereotactic infusion of 0.3 U bacterial collagenase into the right ganglionic eminence of neonatal rats. Briefly, P7 rat pups were secured in a neonatal stereotaxic apparatus and anesthetized with isoflurane (5 % for induction, 3 % for maintenance). A midline scalp incision was made to expose the bregma. A burr hole (1 mm) was drilled at 1.6 mm lateral and 1.5 mm anterior to the bregma. A 10 μL syringe needle (Microliter 701, Hamilton Company) was inserted 2.8 mm below the dura mater, and collagenase was infused at a rate of 1 μL/min using a microinfusion pump (Harvard Apparatus, Hollison, MA). To prevent reflux, the syringe was kept in place for an additional 5 min. The needle was then withdrawn at a rate of 1 mm/min, the burr hole was sealed with bone wax, and the incision was closed with 5-0 silk sutures. After surgery, the animals were placed on a 37 °C heating pad to recover and awaken from anesthesia. Once fully recovered, pups were returned to their dam. Sham-operated animals received identical anesthesia and surgical procedures, followed by an injection of the same volume of phosphate-buffered saline (PBS). The average duration of surgery was approximately 20 min per animal.

### 2.3 Drug administration

Niacin was administered intranasally at 1 h after GMH induction. For the dose-response experiment, niacin was administered intranasally at three different doses (1, 3, and 9 mg/kg) every 12 h for 3 days after GMH to determine the optimal dosage. The dosage of niacin was adapted from previous studies in stroke models, with modifications based on the route of administration [22]. In short-term experiments, niacin was administered intranasally twice daily after GMH, while in long-term experiments, it was administered twice daily for 7 consecutive days following GMH. For the microglial depletion experiment, PLX5622 was administered via intraperitoneal injection once daily starting at birth [23]. Control animals received dimethyl sulfoxide (DMSO) as a vehicle. As previously described, the HCAR2 inhibitor Mepenzolate bromide (MPN), Sirt1 inhibitor Lipo-EX527, Nrf2 inhibitor Lipo-ML385, and DMSO were administered into the ipsilateral ventricle 24 h prior to GMH, while HCAR2 CRISPR plasmid and control CRISPR plasmid were administered into the ipsilateral ventricle 48 h prior to GMH. The injection site was located 1.0 mm posterior and 1.0 mm lateral to the bregma, at a depth of 1.8 mm [21]. Detailed drug information is provided in Supplementary Table S2.

### 2.4 Liposomes administration

Liposomes (Catalog# F10209D, FormuMax Scientific, USA) containing the lipid fluorescent dye Lipo-DHPE (fluorescein DHPE), the SIRT1 inhibitor EX527 (Catalog# E7034, Sigma Aldrich, USA), and the NRF2 inhibitor ML385 (Catalog# SML1833, Sigma Aldrich, USA) were prepared according to the manufacturer's protocol, as previously described [9]. The final concentrations of EX527 and ML385 within the liposomes were determined to be 5 μg/μl using a microplate reader (400 nm, SpectraMax i3x, Molecular Devices, Thermo Fisher Scientific, USA). The liposome-encapsulated inhibitors were subsequently injected into the ventricles of P6 rats of either sex.

### 2.5 Primary microglia culture

Primary microglia were isolated from the cerebral cortex of neonatal Sprague-Dawley rats (postnatal day 0–2) as previously described [24]. Under sterile conditions, cerebral cortices were dissected and transferred into HBSS (Cat# 14175095, Gibco, Carlsbad, USA). After careful removal of the meninges, the tissue was minced and enzymatically digested with 0.25 % trypsin (Cat# 25200072, Gibco, Carlsbad, USA) at 37 °C for 15 min. The digestion was then terminated by adding culture medium containing 10 % FBS/DMEM (Cat# A5256701; Cat# 11965092, Gibco, Carlsbad, USA). The cell suspension was passed through a 75-μm cell strainer, resuspended in 10 % FBS/DMEM medium, and maintained in a humidified incubator (5 % CO_2_, 37 °C) with medium changes every three days. After 10 days, microglia were harvested for in vitro experiments. Oxyhemoglobin (OxyHb) was prepared from hemoglobin (Cat# H2500, Sigma-Aldrich) following the manufacturer's protocol. Primary microglia were incubated with 10 μM OxyHb. Alternatively, red blood cells (RBCs) were isolated and purified from the blood of neonatal rats using Ficoll and subsequently co-cultured with microglia. One hour prior to RBC or hemoglobin stimulation, cells were pretreated with the HCAR2 inhibitor mepenzolate bromide (15 μM). One hour after RBC or hemoglobin exposure, niacin (100 μM) was added to the cultures [20]. The detailed experimental design is illustrated in Supplementary Fig. S1.

### 2.6 Cell viability assay

According to the manufacturer's protocol, cell viability was measured using the Cell Counting Kit-8 (Cat# 96992, Sigma-Aldrich). Absorbance was measured at 450 nm. The results are shown in the Supplementary Fig. S6.

### 2.7 Neurological assessment

To evaluate short- and long-term neurological deficits in neonatal rats, a randomized, blinded, and unbiased protocol was employed [9,21,25]. The righting reflex and negative geotaxis tests were conducted on postnatal days 1–7 following GMH induction to assess early motor dysfunction. From days 22–28 post-GMH, long-term neurological function was assessed using the Morris water maze, Rotarod, and foot-fault tests. The Rotarod test measured motor coordination and balance, the foot-fault test evaluated motor and proprioceptive function, and the Morris water maze assessed spatial learning and memory performance.

#### 2.7.1 Righting reflex test

The time required for each neonatal rat to return from a supine position to a prone, all-limb posture was recorded following GMH. This test was performed once daily for three consecutive days post-GMH, with three trials conducted per day. The average latency from the three trials was calculated for each animal and used for statistical analysis.

#### 2.7.2 Negative geotaxis test

For the negative geotaxis test, each neonatal rat was placed head-downward on a 45° inclined plane. The latency required for the animal to detect its orientation and actively rotate 90° or 180° to face upward was recorded within a 60-s observation window. The test was performed once daily for three consecutive days post-GMH, with three trials per day. The average latency from the three trials was calculated for each animal and used for statistical analysis.

#### 2.7.3 Rotarod test

On day 28 post-GMH, the Rotarod test was conducted to assess motor coordination and balance in rats. Animals were placed on a rotating rod apparatus (7 cm in diameter; Columbus Instruments, Columbus, OH, USA), and the test was initiated at a starting speed of either 5 or 10 RPM. The rotation speed was gradually increased at an acceleration rate of 2 RPM every 5 s. Latency to fall was automatically recorded by an integrated photobeam sensor system (SD Instruments, San Diego, CA, USA).

#### 2.7.4 Foot fault test

On day 28 post-GMH, the foot-fault test was performed to evaluate motor function and proprioception. Rats were placed on a wire grid platform (0.6 × 1.5 m, with 2.5 cm grid spacing) and allowed to walk freely. The number of foot-faults—defined as forelimb or hindlimb slips through the grid—was recorded over a 60-s observation period.

#### 2.7.5 Morris water maze

From days 22–27 post-GMH, the Morris water maze test was conducted to assess spatial learning and memory. Rats were placed in a circular pool (110 cm in diameter) surrounded by fixed visual cues on the inner walls. During the training phase, a submerged escape platform (11 cm in diameter) was located in a designated target quadrant. Each rat underwent 10 training trials per day, and the latency to locate the platform as well as the swim distance were recorded. On the final day, a probe trial was conducted in which the platform was removed. The time spent in the target quadrant was measured to evaluate spatial memory retention. All trials were recorded using an overhead video tracking system (Noldus EthoVision, Noldus, Tacoma, WA, USA), which captured swimming paths and quantified both swim distance and time spent in the target quadrant.

### 2.8 Brain water content

Brain water content was assessed 72 h after GMH induction in neonatal rats. The brain was dissected into the left hemisphere, right hemisphere, and cerebellum, and each region was immediately weighed to determine the wet weight. Tissues were then dried at 100 °C for 72 h to obtain the dry weight. Brain water content was calculated using the following formula:[(wet weight – dry weight)/wet weight] × 100 %.

### 2.9 Nuclear and cytoplasmic fractionation

Nuclear and cytoplasmic proteins were isolated from brain tissue samples collected at various time points after GMH or from sham controls using a nuclear and cytoplasmic extraction kit (Cat# 78835, Thermo Fisher Scientific, USA), according to the manufacturer's protocol.

### 2.10 Western blot

Western blot analysis was performed as previously described [26]. Briefly, at various time points following GMH or in sham controls, neonatal rats were deeply anesthetized with 5 % isoflurane and euthanized by transcardial perfusion with ice-cold 1 × PBS. The right cerebral hemisphere was homogenized using a tissue homogenizer and lysed in RIPA buffer (Cat# sc-24948, Santa Cruz Biotechnology, USA) on ice for 30 min. The lysates were centrifuged at 14,000×*g* for 30 min at 4 °C, and the supernatants were collected. Protein concentrations were determined using a DC Protein Assay Kit (Cat# 5000116, Bio-Rad Laboratories, USA). Equal amounts of protein were separated by SDS-PAGE on 4–20 % gradient gels and transferred onto 0.2 μm or 0.45 μm nitrocellulose membranes. Membranes were blocked with 5 % non-fat milk in TBST for 2 h to prevent non-specific binding, followed by overnight incubation at 4 °C with primary antibodies (Supplementary Table S3). On the following day, membranes were incubated with the appropriate secondary antibodies (Supplementary Table S3) for 1 h at room temperature. Signals were detected using the ECL Plus chemiluminescence system (Cat# RPN2235, Amersham, USA). The relative protein band intensities were quantified using ImageJ software (version 1.53, NIH, USA) and normalized to reference proteins as indicated in Supplementary Table S3. Unedited full-length blots are provided in Supplementary Fig. S9–S13.

### 2.11 Immunofluorescence

Immunofluorescence staining was performed as previously described [25]. Briefly, at 72 h after GMH or in sham controls, neonatal rats were deeply anesthetized with 5 % isoflurane and euthanized by transcardial perfusion with ice-cold 1 × PBS followed by 10 % formalin. Brains were post-fixed in 10 % formalin for 24 h and cryoprotected in a sucrose gradient. The tissue was then embedded in OCT compound and sectioned coronally at 10 μm thickness using a cryostat (LM3050S, Leica Microsystems, Germany). Sections were mounted on adhesive glass slides and permeabilized with 0.1 % Triton X-100 (Sigma-Aldrich, USA) for 30 min. After blocking with 5 % donkey serum (Cat# 017-000-121, Jackson ImmunoResearch, USA) for 1 h at room temperature, sections were incubated overnight at 4 °C with primary antibodies (Supplementary Table S3). On the following day, sections were incubated with fluorophore-conjugated secondary antibodies (Supplementary Table S3) for 1 h at room temperature. Nuclei were counterstained and coverslipped using a DAPI-containing mounting medium (DAPI, Cat# H-1200-10, Vector Laboratories, USA). Fluorescence images were acquired using a fluorescence microscope (DMi8, Leica Microsystems, Germany).

### 2.12 Hemoglobin assay

Hemoglobin content was quantified spectrophotometrically to estimate intracerebral hemorrhage volume, as previously described. Briefly, the right cerebral hemisphere was homogenized in glass tubes containing an equal volume of 1 × PBS. The homogenate was sonicated for 1 min to lyse red blood cells, followed by centrifugation for 30 min to separate the supernatant from the pellet. Drabkin's reagent (Cat# D5941, Sigma-Aldrich, USA) was then added to the supernatant at a 4:1 ratio and incubated at room temperature for 15 min. Absorbance was measured at 540 nm using a spectrophotometer (Genesis 10 UV; Thermo Fisher Scientific, USA), and the hemoglobin concentration (μg/mL) was determined based on a standard curve [21].

### 2.13 ELISA assay

On day 3 post-GMH, brain tissue from the right cerebral hemisphere or cerebrospinal fluid (CSF) was collected from neonatal rats for enzyme-linked immunosorbent assay (ELISA), following the manufacturer's protocol. Levels of IL-1β, TNF-α, IL-6, IL-10, and malondialdehyde (MDA) were quantified using the following kits: IL-1β ELISA kit (Cat# ab255730, Abcam, Cambridge, USA), TNF-α ELISA kit (Cat# ab236712, Abcam, Cambridge, USA), IL-6 ELISA kit (Cat# ab234570, Abcam, Cambridge, USA), IL-10 ELISA kit (Cat# ab214566, Abcam, Cambridge, USA), and MDA assay kit (Cat# MBS162002, MyBioSource, CA, USA).

### 2.14 Dihydroethidium staining

On day 3 post-GMH, brain tissue from the right cerebral hemisphere of neonatal rats was collected for the assessment of reactive oxygen species (ROS) levels. Dihydroethidium (DHE) staining was performed using a DHE staining kit (Cat# D11347, Thermo Fisher Scientific, USA) according to the manufacturer's protocol to evaluate total ROS levels. Fluorescence images were acquired using a fluorescence microscope (DMi8, Leica Microsystems, Germany).

### 2.15 Iron staining

On days 3 and 7 post-GMH, ferric iron (Fe^3+^) deposition in the perihematomal region was assessed using an iron staining kit (Cat# ab150674, Abcam, Cambridge, USA) according to the manufacturer's protocol. Stained sections were visualized using an Olympus BX51 microscope.

### 2.16 Fluoro-Jade C staining

On day 3 post-GMH, neuronal degeneration in the perihematomal region was assessed using a Fluoro-Jade C (FJC)(Cat# 76264-660, Biosensis, USA) staining kit according to the manufacturer's protocol. Fluorescent images were acquired using a fluorescence microscope (DMi8, Leica Microsystems, Germany).

### 2.17 Nissl staining

On day 28 post-GMH, rats were deeply anesthetized with 5 % isoflurane and euthanized by transcardial perfusion with ice-cold 1 × PBS followed by 10 % formalin. Brains were post-fixed in 10 % formalin for 24 h and cryoprotected in a sucrose gradient. The processed brains were embedded in OCT compound and coronally sectioned at 20 μm thickness using a cryostat. Sections were mounted on adhesive glass slides and stained with 0.5 % cresyl violet acetate (Cat# C5042, Sigma-Aldrich, USA). Stained sections were visualized using an Olympus BX51 microscope. Ventricular volume, white matter loss, and cortical thickness were measured as previously described to quantify GMH-induced brain injury.

### 2.18 Molecular docking

Protein sequences were retrieved from the UniProt database, and protein–protein interaction structures were predicted using AlphaFold3. The predicted protein complex structures were visualized in PyMOL (version 2.3.4) to identify potential hydrogen bond–forming amino acid residue pairs between interacting proteins. Binding affinity of the predicted complexes was subsequently evaluated using the PRODIGY tool based on calculated binding free energies.

### 2.19 Statistical analysis

All statistical analyses and graph generation were performed using GraphPad Prism 10.0. Data are presented as mean ± standard deviation (mean ± SD). One-way analysis of variance (ANOVA) followed by Tukey's post hoc test was used to assess differences among multiple groups. Neurobehavioral test data were analyzed using two-way ANOVA. *P* < 0.05 was considered statistically significant.

## 3 Results

### 3.1 Time course and spatial expression of HCAR2 in the brain after GMH

**Fig. 1:**
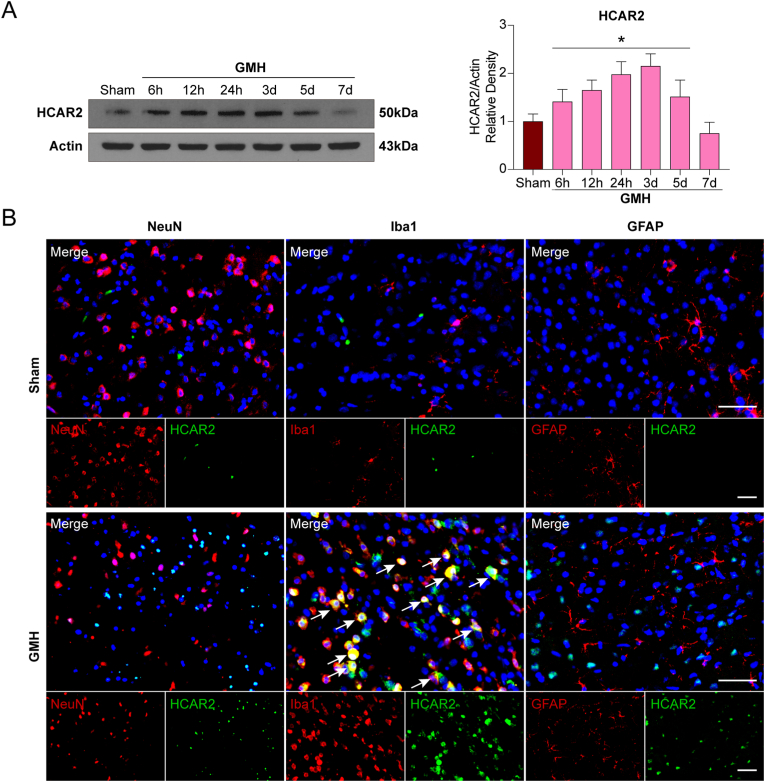
**Temporal expression and cellular localization of hydroxycarboxylic acid receptor 2 (HCAR2) in the brain after germinal matrix hemorrhage (GMH). A**, Representative Western blot images and quantitative analyses of HCAR2 at 6, 12, and 24 h and 3, 5, and 7 days after GMH. Values are expressed as mean ± SD. n = 6 per group. ∗P < 0.05 vs. Sham. **B**, Representative images of immunofluorescence staining showing the colocalization of HCAR2 (green) with neurons, microglia, and astrocytes (NeuN [neuronal nuclei], Iba1 [ionized calcium-binding adaptor molecule 1], GFAP [glial fibrillary acidic protein], red) in the pups with or without GMH. Scale bar = 50 μm. n = 4 per group.

A time-course analysis was performed to evaluate the temporal expression pattern of HCAR2 in the ipsilateral (right) cerebral hemisphere following GMH. Western blot results revealed that HCAR2 expression began to increase at 6 h post-GMH, reached a peak on day 3, and subsequently declined by days 5 and 7 (P < 0.05; Fig. 1A**)**. To determine the cellular localization of HCAR2, double immunofluorescence staining was conducted on day 3 after GMH. HCAR2 was found to be predominantly colocalized with microglia in the periventricular region (Fig. 1B). In contrast, minimal HCAR2 expression was observed in neurons, astrocytes, or microglia in the Sham group (Fig. 1B). These data demonstrate that HCAR2 is dynamically upregulated after GMH and is predominantly localized to microglia in the periventricular region.

### 3.2 Intranasal administration of niacin improves short-term outcomes and long-term neurological function after GMH

**Fig. 2:**
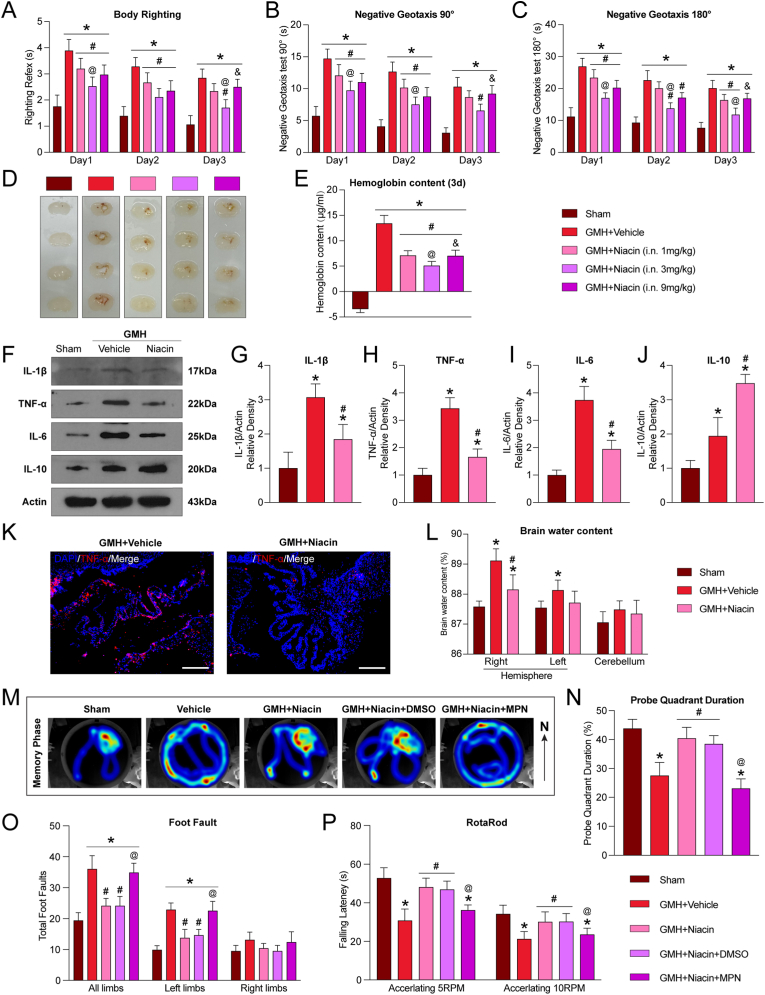
**Intranasal administration of niacin improved short-term neurological outcomes and ameliorated long-term neurobehavioral deficits after germinal matrix hemorrhage (GMH). A-C**, GMH-induced neurological deficits were evaluated by Righting reflex and Geotaxis reflex tests. **D-E**, Hemoglobin levels were measured at 72 h after GMH induction. Niacin at all tested doses significantly reduced hemoglobin content at 72 h after GMH. Values are expressed as mean ± SD. One-way ANOVA followed by Tukey's test. n = 6 per group. ∗P < 0.05 vs Sham, #P < 0.05 vs GMH + Vehicle, @P < 0.05 vs GMH + Niacin (1 mg/kg), &P < 0.05 vs GMH + Niacin (3 mg/kg). **K**, Immunofluorescence staining showed that niacin treatment significantly reduced TNF-α immunoreactivity in the choroid plexus compared with the vehicle-treated group. Scale bar = 200 μm. n = 4 per group. **L**, Brain water content on day 3 after GMH. n = 6 per group. **F-J**, Western blot analysis showed that niacin reduced the levels of pro-inflammatory cytokines IL-1β, TNF-α, and IL-6 in the brain, while increasing the expression of the anti-inflammatory cytokine IL-10. n = 6 per group. **M-N**, Representative heat map of the probe test and probe quadrant duration on days 22–27 after GMH. n = 8 per group. **O–P**, Foot-fault and rotarod tests were performed on day 28 after GMH. n = 8 per group. Values are expressed as mean ± SD. One-way ANOVA followed by Tukey's test. n = 6/8 per group. ∗P < 0.05 vs Sham, #P < 0.05 vs GMH + Vehicle, @P < 0.05 vs. GMH + Niacin or GMH + Niacin + dimethyl sulfoxide (DMSO). MPN, Mepenzolate bromide.

To explore the translational therapeutic potential of niacin, pups received intranasal doses of 1, 3, or 9 mg/kg at 1-h post-GMH, followed by dosing every 12 h for 3 days. At 24 h, vehicle-treated pups showed significant neurological deficits compared to the sham group (P<0.05; Fig. 2A–C). Treatment with 3 mg/kg niacin significantly improved performance in the body righting and negative geotaxis tests over 3 days (P<0.05; Fig. 2A–C). At 72 h, all niacin doses reduced brain hemoglobin content, with the 3 mg/kg dose showing the greatest effect (P<0.05; Fig. 2D and E). Thus, 3 mg/kg was selected for subsequent studies.

To further assess the short-term neuroprotective effects of niacin, FJC staining was performed to detect neuronal degeneration surrounding the hematoma. On day 3 post-GMH, FJC-positive cells in the perihema region were significantly increased but markedly reduced by niacin treatment (P<0.05; Supplementary Fig. S2A–B). Given the known anti-inflammatory properties of niacin, we next evaluated cytokine levels. GMH increased pro-inflammatory cytokines (IL-1β, TNF-α, IL-6), with a slight rise in the anti-inflammatory cytokine IL-10, whereas niacin reduced pro-inflammatory cytokines and further enhanced IL-10 expression (P<0.05; Fig. 2F–J). Immunofluorescence confirmed reduced TNF-α expression in the choroid plexus following niacin treatment (Fig. 2K), consistent with Western blot results. Additionally, niacin significantly reduced brain water content in the ipsilateral hemisphere, with no effect on the contralateral side or cerebellum (P<0.05; Fig. 2L), indicating reduced regional edema. Together, these results demonstrate that niacin promotes hematoma clearance and suppresses secondary injury events, including hemoglobin accumulation, neuroinflammation, and edema formation, thereby improving short-term neurological outcomes after GMH.

To assess the long-term therapeutic effects of niacin, intranasal administration was initiated 1 h after GMH and continued for 7 days. Niacin significantly improved postnatal weight gain compared to the vehicle group (P<0.05; Supplementary Fig. S3A). From days 21–27 post-GMH, long-term neurological function was evaluated using the Morris water maze, rotarod, and foot-fault tests. In the Morris water maze, vehicle-treated rats showed longer escape latencies, greater swim distances, and less time in the target quadrant compared to Sham controls (P<0.05; Fig. 2M and N; Supplementary Fig. S3B–D). Niacin significantly improved these deficits, while co-treatment with MPN abolished the benefits (P<0.05; Fig. 2M and N; Supplementary Fig. S3B–D). In the rotarod test, vehicle-treated rats had shorter latency to fall at both 5 and 10 rpm, whereas niacin significantly increased latency, indicating better motor coordination and balance (P<0.05; Fig. 2O). This effect was reversed by MPN (P<0.05; Fig. 2O). Similarly, in the foot-fault test, niacin reduced the number of foot faults, reflecting improved motor and proprioceptive function—an effect also blocked by MPN (P<0.05; Fig. 2P).

Collectively, these findings indicate that niacin confers both short- and long-term neuroprotection after GMH, potentially via HCAR2 signaling, warranting further mechanistic investigation.

### 3.3 Niacin enhanced hematoma resolution after GMH

**Fig. 3:**
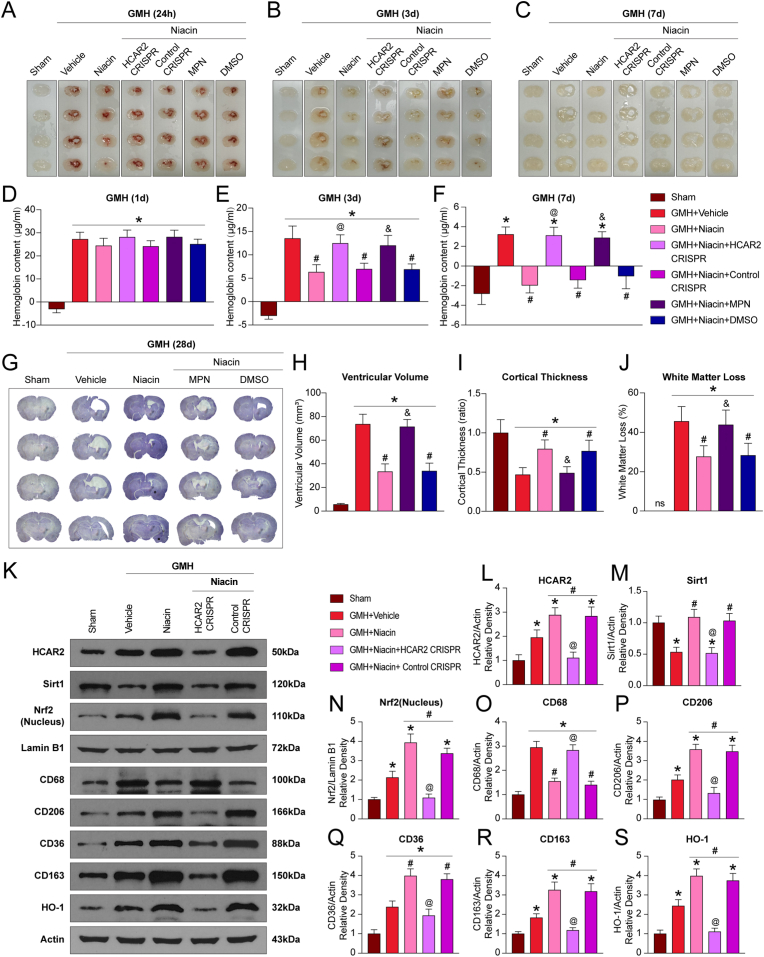
**Niacin promoted hematoma clearance after germinal matrix hemorrhage (GMH). A-C**, Representative brain sections at 24 h, 72 h, and 7 d after GMH. **D-F**, Quantification of hematoma content. Values are expressed as mean ± SD. One-way ANOVA followed by Tukey's test. n = 6 per group. ∗P < 0.05 vs. Sham; #P < 0.05 vs. GMH + Vehicle; @P < 0.05 vs. GMH + Niacin or GMH + Niacin + Control CRISPR; &P < 0.05 vs. GMH + Niacin or GMH + Niacin + dimethyl sulfoxide (DMSO). **G-J**, Quantitative analysis of Nissl-stained brain sections showed that niacin significantly reduced ventricular volume and white matter loss, while increasing cortical thickness in GMH pups. n = 8 per group. Values are expressed as mean ± SD. One-way ANOVA followed by Tukey's test. n = 8 per group. ∗P < 0.05 vs Sham, #P < 0.05 vs GMH + Vehicle, @P < 0.05 vs. GMH + Niacin or GMH + Niacin + dimethyl sulfoxide (DMSO). **K**, Representative Western blot bands showing HCAR2, Sirt1, Nrf2(Nucleus), CD68, CD206, CD36, CD163, and HO-1 expression. **L-S**, Quantification of Western blot results. Values are expressed as mean ± SD. One-way ANOVA followed by Tukey's test. n = 6 per group. ∗P < 0.05 vs. Sham; #P < 0.05 vs. GMH + Vehicle; @P < 0.05 vs. GMH + Niacin or GMH + Niacin + Control CRISPR. MPN, Mepenzolate bromide.

To assess the short-term effects of niacin on hematoma clearance, brain hemoglobin content was measured at 24 h, 3 days, and 7 days after GMH. At 24 h, there were no significant differences among groups treated with niacin, HCAR2 CRISPR, or MPN (P>0.05; Fig. 3A–F). By days 3 and 7, hemoglobin levels were significantly reduced in the niacin group compared to vehicle, whereas this reduction was abolished by either HCAR2 CRISPR or MPN (P<0.05; Fig. 3A–F). Notably, hematoma volume on day 3 was already decreased or showed a strong downward trend in niacin-treated animals and became more pronounced by day 7. Both HCAR2 CRISPR and MPN negated this effect (Fig. 3A–C). Neurological assessments conducted at 24 h, 3 days, and 7 days demonstrated that niacin significantly improved functional outcomes compared to vehicle treatment, while these improvements were prevented by HCAR2 CRISPR and MPN (P<0.05; Supplementary Fig. S4A–C).

To evaluate the long-term impact of niacin on hematoma resolution and the development of secondary hydrocephalus, ventricular volume was measured on day 28 using Nissl staining. Compared with the sham group, vehicle-treated rats exhibited significantly enlarged ventricles (P<0.05; Fig. 3G and H). Niacin treatment markedly reduced ventricular volume, suggesting suppression of hydrocephalus progression (P<0.05; Fig. 3G and H). Furthermore, vehicle-treated animals showed substantial white matter loss and cortical thinning, both of which were significantly alleviated by niacin (P<0.05; Fig. 3G–I, J). These neuroprotective effects were diminished in the presence of MPN (P<0.05; Fig. 3G–I, J).

To determine whether niacin promotes hematoma clearance by modulating microglial polarization via HCAR2, we assessed the expression of relevant molecular markers. Niacin suppressed the M1 marker CD68 while upregulating HCAR2, Sirt1, and the M2 marker CD206(P<0.05; Fig. 3K–S); these effects were significantly reversed by HCAR2 knockdown(P<0.05; Fig. 3K–S). In addition, niacin markedly increased the expression of Nrf2, CD36, CD163, and HO-1, whereas HCAR2 CRISPR abolished these upregulations(P<0.05; Fig. 3K–S). Immunofluorescence analysis further showed that both HCAR2 CRISPR and MPN significantly reduced the colocalization of HCAR2 with Iba1(P<0.05; Supplementary Fig. S5A–C).

Collectively, these findings suggest that niacin may facilitate microglial M2 polarization and enhance phagocytic activity through an HCAR2-dependent mechanism, thereby accelerating hematoma clearance after GMH.

### 3.4 Niacin enhances microglial phagocytosis and promotes M2 microglial accumulation in the periventricular region after GMH

Given the observed reduction in hemoglobin content and hematoma volume following niacin treatment, we next investigated its role in microglia-mediated hematoma clearance. CD36 and CD163, two class B scavenger receptors primarily expressed on M2-polarized microglia surrounding the hematoma core, are essential for erythrophagocytosis and hemoglobin clearance, respectively [24].

**Fig. 4:**
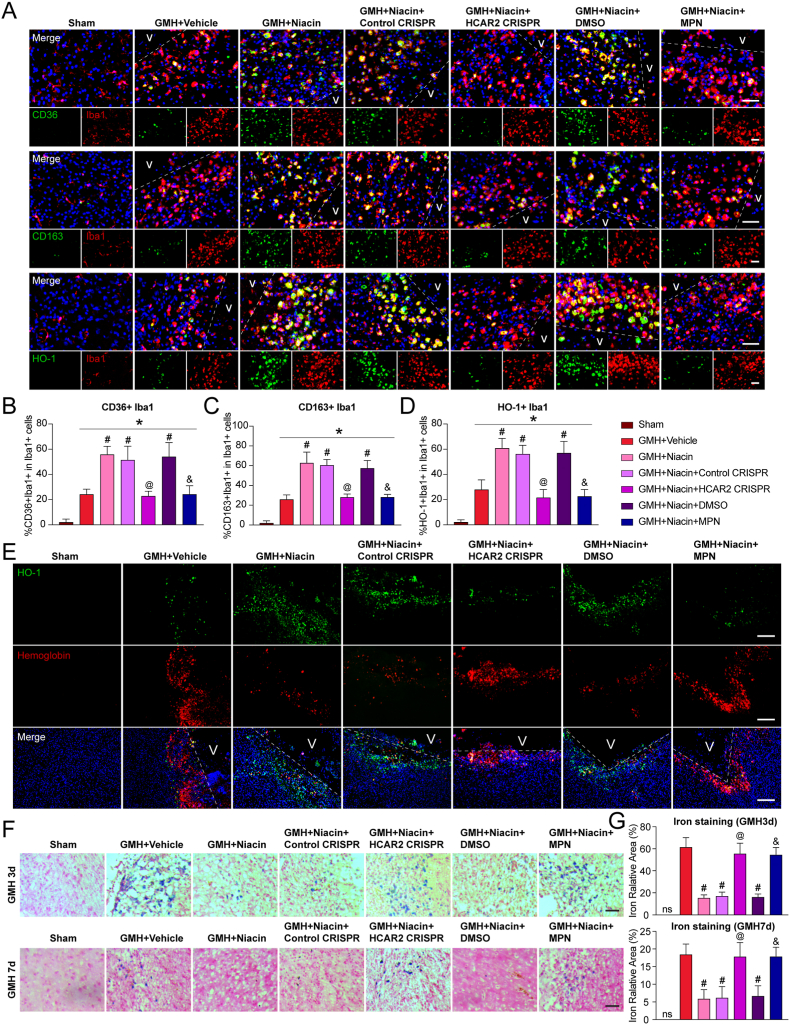
**Niacin promoted hematoma clearance by increasing M2 microglia in the periventricular region after germinal matrix hemorrhage (GMH). A,** Representative immunofluorescence images showing CD36^+^/CD163^+^/HO-1^+^microglia (CD36^+^/CD163^+^/HO-1^+^, green; Iba1^+^ [ionized calcium-binding adaptor molecule 1], red) in the periventricular region after GMH. Scale bar = 50 μm. n = 4 per group. **B-D**, Quantitative analysis of CD36^+^/CD163^+^/HO-1^+^ microglia. **E**, Representative immunofluorescence images showing colocalization of HO-1 with hemoglobin in the periventricular region after GMH. Scale bar = 200 μm. n = 4 per group. **F-G**, Representative images of Iron staining and quantitative analysis. Scale bar = 50 μm. n = 4 per group. Values are expressed as mean ± SD. One-way ANOVA followed by Tukey's test. n = 4 per group. ∗P < 0.05 vs. Sham; #P < 0.05 vs. GMH + Vehicle; @P < 0.05 vs. GMH + Niacin or GMH + Niacin + Control CRISPR; &P < 0.05 vs. GMH + Niacin or GMH + Niacin + dimethyl sulfoxide (DMSO). MPN, Mepenzolate bromide. V, Ventricles.

At 3 days post-GMH, the number of CD36^−^and CD163-positive microglia in the periventricular region was significantly increased in the niacin-treated group compared to vehicle controls, whereas this increase was markedly attenuated by both HCAR2 CRISPR and MPN (P<0.05; Fig. 4A–C). Following phagocytosis, microglia degrade the internalized hematoma via lysosomal pathways [26]. Extracellular hemoglobin undergoes oxidation and dimerization, leading to the release of free heme, which is subsequently metabolized by HO-1 into iron, carbon monoxide (CO), and biliverdin [6]. To determine whether niacin facilitates this process, we examined HO-1 expression in microglia. In the periventricular region, HO-1-positive microglia were rarely observed in the sham group, while a modest increase was detected in vehicle-treated rats (P<0.05; Fig. 4A–D). In contrast, niacin treatment significantly elevated the number of HO-1-positive microglia, and this effect was markedly reduced by HCAR2 CRISPR and MPN (P<0.05; Fig. 4A–D). Co-immunostaining of HO-1 and hemoglobin revealed clear colocalization in the periventricular region (Fig. 4E). Compared to the substantial hemoglobin accumulation observed in the vehicle group, niacin treatment led to extensive hemoglobin degradation by HO-1(Fig. 4E). This clearance effect was significantly diminished following HCAR2 CRISPR or MPN administration (Fig. 4E).

Iron, a major byproduct of hemoglobin degradation, contributes to oxidative stress, lipid peroxidation, and cytotoxicity, thereby exacerbating brain injury after GMH. Iron staining at 3 and 7 days post-GMH showed significantly reduced iron deposition in the periventricular region following niacin treatment compared with vehicle (P<0.05; Fig. 4F and G). However, iron accumulation was elevated in animals treated with HCAR2 CRISPR or MPN (P<0.05; Fig. 4F and G).

In summary, niacin activates HCAR2 to drive microglial polarization toward the M2 phenotype, thereby enhancing CD36/CD163/HO-1–mediated erythrophagocytosis and hemoglobin degradation, and significantly suppressing iron accumulation surrounding the hematoma.

### 3.5 Niacin attenuates neuroinflammation and oxidative stress after GMH

Hematoma and its degradation products can trigger microglia-mediated neuroinflammation, while reactive oxygen species (ROS) rapidly accumulate in the brain following intracerebral hemorrhage, contributing to persistent oxidative stress—both processes critically exacerbate neurological dysfunction [27]. Given that niacin promotes microglial polarization toward the M2 phenotype and upregulates Nrf2—a key transcription factor known to attenuate inflammation and oxidative stress—we next evaluated its effects on microglia-mediated neuroinflammatory and oxidative responses.

**Fig. 5:**
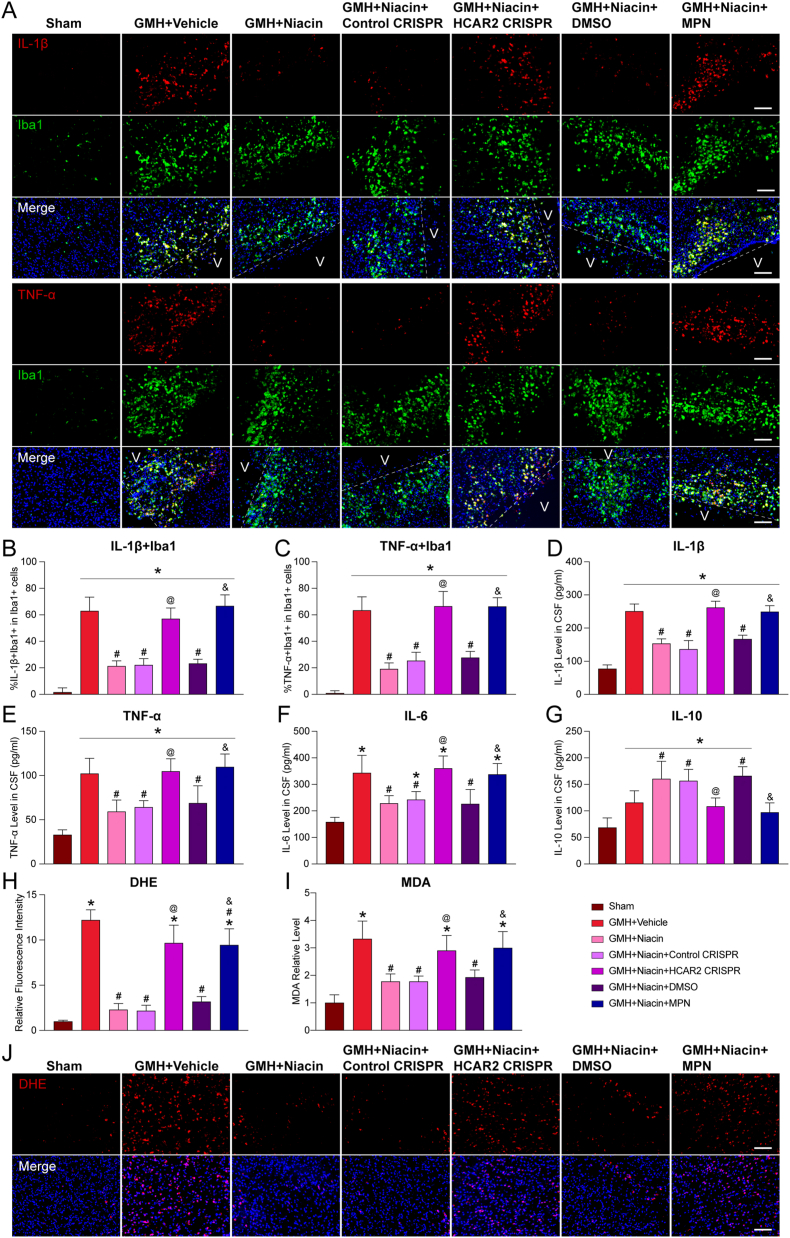
**Niacin significantly attenuated GMH-induced neuroinflammation and oxidative stress. A**, Representative immunofluorescence images showing IL-1β^+^/TNF-α^+^microglia (IL-1β^+^/TNF-α^+^, green; Iba1^+^ [ionized calcium-binding adaptor molecule 1], red) in the periventricular region after GMH. Scale bar = 100 μm. n = 4 per group. **B–C**, Quantitative analysis of IL-1β^+^/TNF-α^+^ microglia. **D-G**, The concentrations of IL-1β, TNF-α, IL-6, and IL-10 in the cerebrospinal fluid on day 3 after GMH were analyzed using ELISA. n = 6 per group. **H/J**, Representative images of DHE staining and quantitative analysis. Scale bar = 100 μm. n = 4 per group. **I**, Quantitative ELISA analysis of brain MDA levels on day 3 after GMH. n = 6 per group. Values are expressed as mean ± SD. One-way ANOVA followed by Tukey's test. n = 4/6 per group. ∗P < 0.05 vs. Sham; #P < 0.05 vs. GMH + Vehicle; @P < 0.05 vs. GMH + Niacin or GMH + Niacin + Control CRISPR; &P < 0.05 vs. GMH + Niacin or GMH + Niacin + dimethyl sulfoxide (DMSO). MPN, Mepenzolate bromide. V, Ventricles.

On day 3 post-GMH, immunofluorescence staining revealed a marked increase in IL-1β- and TNF-α–positive microglia in the periventricular region, indicating a robust neuroinflammatory response (P<0.05; Fig. 5A–C). Niacin treatment significantly reduced the number of cytokine-positive microglia, while this anti-inflammatory effect was notably diminished by HCAR2 CRISPR and MPN administration (P<0.05; Fig. 5A–C). To further assess cytokine secretion, cerebrospinal fluid (CSF) was collected and analyzed by ELISA. Compared with the vehicle group, niacin significantly decreased levels of pro-inflammatory cytokines IL-1β, TNF-α, and IL-6, and increased the anti-inflammatory cytokine IL-10 (P<0.05; Fig. 5D–G). These regulatory effects were substantially disrupted by HCAR2 CRISPR and MPN (P<0.05; Fig. 5D–G), consistent with the immunofluorescence findings. We next examined oxidative stress in the ipsilateral hemisphere on day 3 post-GMH using dihydroethidium (DHE) staining to detect ROS and MDA assays to assess lipid peroxidation. Compared with Sham, the vehicle group exhibited significantly elevated DHE fluorescence, reflecting severe oxidative damage (P<0.05; Fig. 5H–J). Niacin treatment markedly reduced ROS accumulation, and this antioxidant effect was markedly weakened following HCAR2 CRISPR or MPN treatment (P<0.05; Fig. 5H–J). Consistently, MDA levels were significantly reduced in the niacin group and elevated in the presence of HCAR2 CRISPR and MPN (P<0.05; Fig. 5I).

Overall, niacin demonstrated potent anti-inflammatory and antioxidative effects in the context of GMH.

### 3.6 The therapeutic effect of niacin depends on the presence of microglia

**Fig. 6:**
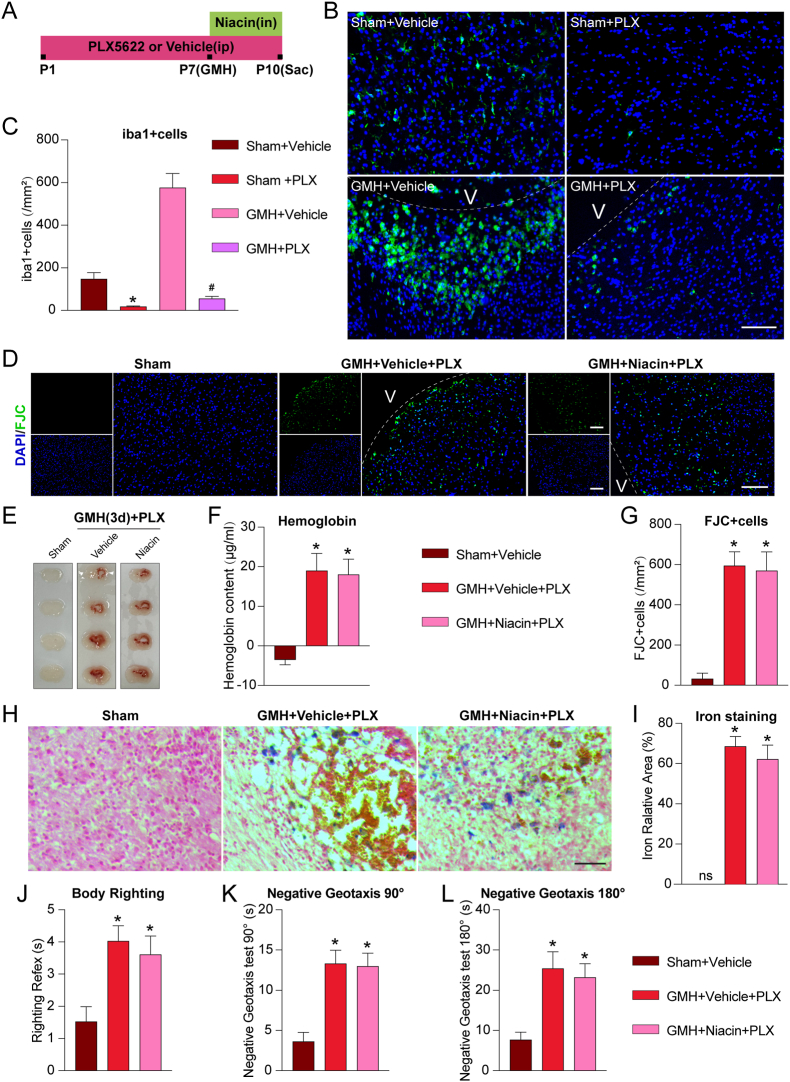
**PLX5622-mediated microglial depletion attenuated the effects of niacin in GMH pups. A**, Schematic illustration of the PLX5622 administration timeline for microglial depletion. **B–C**, Representative immunofluorescence images of Iba1^+^ microglia (green) in the ventricular region, and quantitative analysis of Iba1^+^ microglial density. Scale bar = 100 μm. n = 4 per group. **D/G**, Representative images of FJC staining and quantitative analysis. Scale bar = 100 μm. n = 4 per group. **E-F**, Representative brain sections and quantification of hematoma content at 3 days after GMH. n = 6 per group. **H-J**, Representative images of Iron staining and quantitative analysis. Scale bar = 50 μm. n = 4 per group. **J-L**, Niacin-mediated improvement in short-term neurological function after GMH in pups is microglia-dependent. n = 6 per group. Values are expressed as mean ± SD. One-way ANOVA followed by Tukey's test. n = 4/6 per group. ∗P < 0.05 vs Sham, #P < 0.05 vs GMH + Vehicle + PLX. PLX, PLX5622. V, Ventricles.

To determine whether the therapeutic effects of niacin are mediated primarily through resident microglia rather than peripherally derived monocyte–macrophages, we employed the CSF1R inhibitor PLX5622 to selectively deplete microglia in neonatal rat brains [23]. Beginning on postnatal day 1 (P1), PLX5622 was administered via daily intraperitoneal injection until euthanasia at P10 (Fig. 6A). On day 3 after GMH (P10), immunofluorescence staining confirmed a significant reduction in the number of microglia within the periventricular region in both Sham and GMH pups (P<0.05; Fig. 6B and C).

We next evaluated whether niacin could promote hematoma clearance in the absence of microglia. Hemoglobin content in the GMH + Vehicle + PLX and GMH + Niacin + PLX groups showed no significant difference (P>0.05; Fig. 6E and F), suggesting that niacin's effect was abolished by microglial depletion. Consistent with these findings, iron staining revealed no significant difference in perihema iron accumulation between the GMH + Vehicle + PLX and GMH + Niacin + PLX groups (P>0.05; Fig. 6H and I), indicating that niacin failed to reduce iron burden in the absence of microglia. Short-term neurological outcomes were assessed using the body righting and negative geotaxis tests. While the GMH + Niacin + PLX group performed slightly better than the GMH + Vehicle + PLX group, the differences were not statistically significant (P>0.05; Fig. 6J–L). Similarly, FJC staining showed a modest reduction in neuronal degeneration in the niacin-treated group compared with vehicle, but again without statistical significance (P>0.05; Fig. 6D–G).

Together, these results demonstrate that the neuroprotective effects of niacin depend on the presence of microglia and are largely diminished following their depletion.

### 3.7 Niacin enhances erythrophagocytosis in primary microglia in vitro

**Fig. 7:**
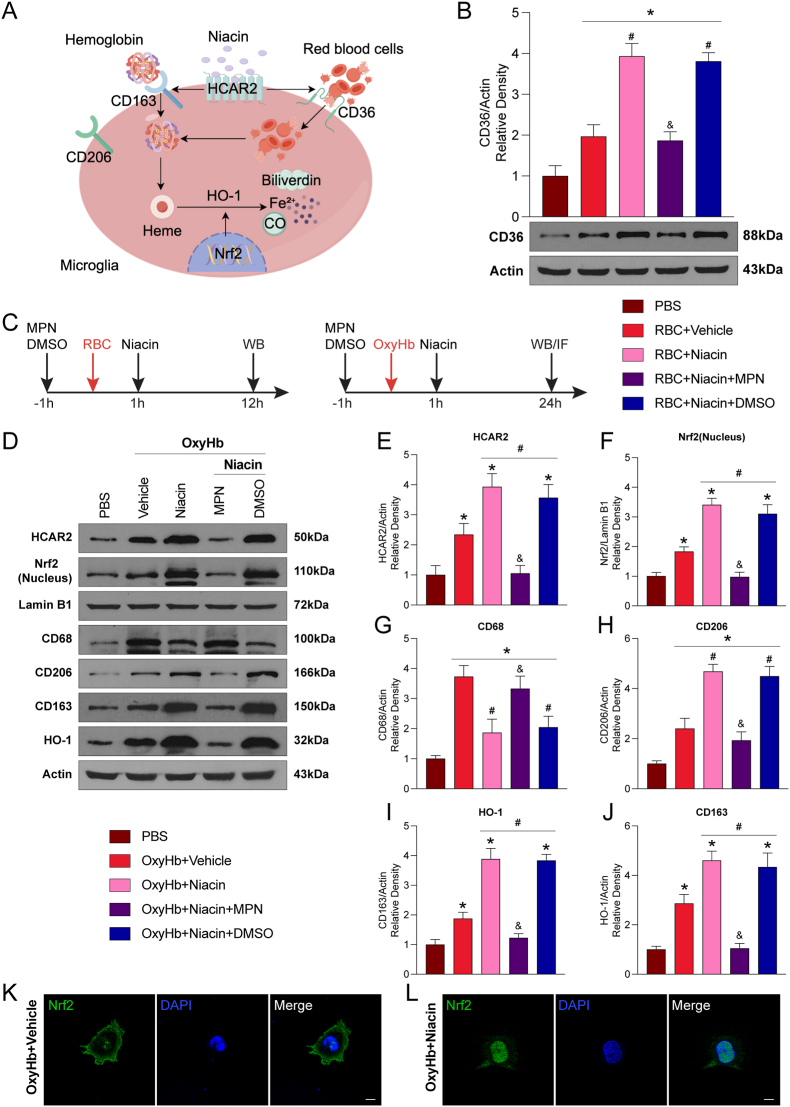
**Niacin promoted M2 polarization of primary microglia and enhanced their ability to phagocytose erythrocytes and hemoglobin. A/C**, Schematic diagram illustrating the mechanism by which niacin promotes microglial phagocytosis of erythrocytes and hemoglobin, and the timeline of the experimental design. **B**, Western blot analysis showed that niacin upregulated CD36 protein levels in primary microglia. n = 6 per group. Values are expressed as mean ± SD. One-way ANOVA followed by Tukey's test. n = 6 per group. ∗P < 0.05 vs. PBS; #P < 0.05 vs. RBC + Vehicle; &P < 0.05 vs. RBC + Niacin or RBC + Niacin + dimethyl sulfoxide (DMSO). **D**, Representative Western blot bands showing HCAR2, Nrf2(Nucleus), CD68, CD206, CD163, and HO-1 expression. n = 6 per group. **E-J**, Quantification of Western blot results. Values are expressed as mean ± SD. One-way ANOVA followed by Tukey's test. n = 6 per group. ∗P < 0.05 vs. PBS; #P < 0.05 vs. OxyHb + Vehicle; &P < 0.05 vs. OxyHb + Niacin or OxyHb + Niacin + dimethyl sulfoxide (DMSO). **K-L**, Representative immunofluorescence images showing laser confocal colocalization of Nrf2 with the nucleus in primary microglia. Scale bar = 10 μm. n = 4 per group. MPN, Mepenzolate bromide.

Given that the hematoma clearance effect of niacin depends primarily on microglia, we assessed the capacity of primary microglia to phagocytose red blood cells (RBCs) in vitro. Since microglial recognition and engulfment of RBCs relies on CD36, we first examined CD36 protein expression by Western blot [24]. Compared with the PBS group, RBC stimulation in the vehicle group slightly increased CD36 expression (P<0.05; Fig. 7B). In contrast, niacin treatment markedly upregulated CD36 levels, an effect that was abolished by MPN (P<0.05; Fig. 7B), indicating that niacin enhances erythrophagocytosis via CD36 in an HCAR2-dependent manner.

To further investigate how niacin influences hemoglobin uptake and degradation by M2-polarized microglia, we assessed the expression of key regulatory molecules by Western blot. OxyHb stimulation significantly upregulated CD68 (an M1 marker), with only a slight increase in CD206 (an M2 marker) compared to PBS controls (P<0.05; Fig. 7D–J). Niacin treatment promoted a shift toward an anti-inflammatory M2 phenotype, as evidenced by reduced CD68 and elevated CD206 expression (P<0.05; Fig. 7D–J). Additionally, niacin significantly increased the expression of HCAR2, Nrf2, CD163, and HO-1 compared to vehicle, while co-treatment with MPN attenuated these effects (P<0.05; Fig. 7D–J).

In summary, these in vitro findings confirm that niacin enhances microglial phagocytic capacity through activation of HCAR2 signaling.

### 3.8 Niacin enhances hematoma clearance and neurological recovery via the HCAR2/SIRT1/Nrf2 pathway

To determine whether the SIRT1/Nrf2 signaling pathway in microglia mediates the protective effects of niacin after GMH, we administered liposome-encapsulated SIRT1 inhibitor (Lipo-EX527) or Nrf2 inhibitor (Lipo-ML385) into the cerebral ventricles of neonatal rats 24 h before GMH induction, targeting microglial populations (Supplementary Fig. S7A).

**Fig. 8:**
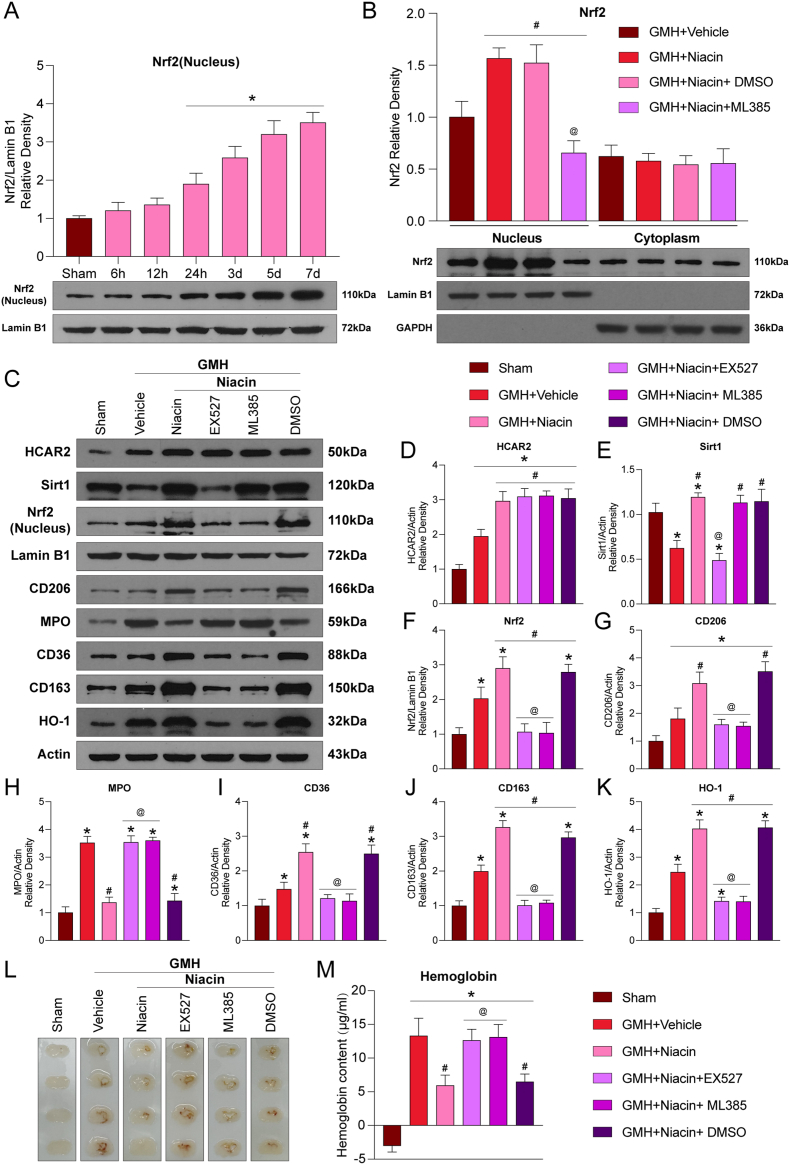
**Niacin enhanced microglial phagocytosis of hematoma after GMH through the HCAR2/SIRT1/Nrf2 signaling pathway. A**, Representative Western blot images and quantitative analyses of Nrf2(Nucleus) at 6, 12, and 24 h and 3, 5, and 7 days after GMH. Values are expressed as mean ± SD. n = 6 per group. ∗P < 0.05 vs. Sham. **B**, Representative Western blot bands showing nuclear and cytoplasmic expression of Nrf2, and corresponding quantitative analysis. Values are expressed as mean ± SD. One-way ANOVA followed by Tukey's test. n = 6 per group. #P < 0.05 vs. GMH + Vehicle; @P < 0.05 vs. GMH + Niacin or GMH + Niacin + dimethyl sulfoxide (DMSO). **C**, Representative Western blot bands showing HCAR2, Sirt1, Nrf2(Nucleus), CD206, MPO, CD36, CD163, and HO-1 expression. **D-K**, Quantification of Western blot results. n = 6 per group. **L-M**, Representative brain sections and quantification of hematoma content. n = 6 per group. Values are expressed as mean ± SD. One-way ANOVA followed by Tukey's test. n = 6 per group. ∗P < 0.05 vs. Sham; #P < 0.05 vs. GMH + Vehicle; @P < 0.05 vs. GMH + Niacin or GMH + Niacin + dimethyl sulfoxide (DMSO).

Nrf2 has been widely recognized as a pivotal neuroprotective transcription factor in the context of hemorrhagic stroke [28].We first examined the temporal profile of Nrf2 expression by Western blot, which revealed a progressive increase in Nrf2 levels following GMH (P<0.05; Fig. 8A). As Nrf2 exerts its function predominantly in the nucleus, we performed immunofluorescence staining in primary microglia to assess its subcellular localization. OxyHb stimulation induced partial nuclear translocation of Nrf2, whereas niacin treatment markedly enhanced Nrf2 nuclear accumulation (Fig. 7K and L). Consistently, nuclear-cytoplasmic fractionation in brain tissue showed significantly higher nuclear Nrf2 levels after GMH, which were further elevated by niacin and suppressed by Lipo-ML385 (P<0.05; Fig. 8B). Cytoplasmic Nrf2 expression remained unchanged across groups (P>0.05; Fig. 8B).

To identify upstream regulators of Nrf2, molecular docking analysis revealed strong predicted binding affinities between Nrf2 and SIRT1 (−8.5 kcal/mol), and between SIRT1 and HCAR2 (−9.7 kcal/mol), suggesting a sequential interaction (Supplementary Fig. S7B–C). We next assessed protein expression within the HCAR2/SIRT1/Nrf2 pathway. Compared with Sham, the vehicle group exhibited increased expression of HCAR2, Nrf2, CD206, CD36, CD163, and HO-1, but decreased SIRT1 levels (P<0.05; Fig. 8C–K). Niacin significantly upregulated these proteins compared to vehicle, while reducing the expression of the neutrophil marker MPO (P<0.05; Fig. 8C–K). Notably, neither Lipo-EX527 nor Lipo-ML385 affected HCAR2 expression (P>0.05; Fig. 8C and D). However, SIRT1 expression was significantly reduced by Lipo-EX527 but not by Lipo-ML385, while Nrf2 levels were suppressed by Lipo-EX527 but not vice versa, indicating that SIRT1 acts upstream of Nrf2 (P<0.05; Fig. 8C–F). Furthermore, both inhibitors decreased the expression of CD206, CD36, CD163, and HO-1, and increased MPO expression (P<0.05; Fig. 8C–K), suggesting impaired microglial phagocytosis and enhanced inflammation.

To assess the involvement of SIRT1/Nrf2 signaling in hematoma clearance, hemoglobin levels were measured in the ipsilateral hemisphere. Lipo-EX527 or Lipo-ML385 treatment significantly increased hemoglobin content compared to niacin or niacin + DMSO groups (P<0.05; Fig. 8L and M). Immunofluorescence confirmed greater hemoglobin accumulation in the periventricular region (Supplementary Fig. S8A), indicating that niacin promotes hematoma clearance via microglial SIRT1/Nrf2 signaling. Similarly, MPO + neutrophil infiltration, which was reduced by niacin, was increased following inhibitor treatment (Supplementary Fig. S8B). Behavioral assessments further demonstrated that niacin significantly improved neurological performance in the body righting and negative geotaxis tests, whereas Lipo-EX527 and Lipo-ML385 treatment prolonged response times and exacerbated neurological deficits (P<0.05; Supplementary Fig. S8C–E).

In summary, these findings underscore the critical role of microglial HCAR2/SIRT1/Nrf2 signaling in mediating neuroprotection after GMH.

## 4 Discussion

Germinal matrix hemorrhage (GMH) is a common and devastating form of neonatal stroke with limited effective treatment options. The primary therapeutic goals after GMH are to promote hematoma resolution and mitigate neurological deficits [29]. In this study, we propose a safe, non-invasive intervention that targets early pathological events of GMH to enhance endogenous hematoma clearance and attenuate secondary brain injury. We found that HCAR2 expression is dynamically upregulated after GMH, predominantly in periventricular microglia. Intranasal administration of niacin activates HCAR2, inhibits M1 polarization, and promotes M2-like phenotype transformation of microglia, thereby suppressing pro-inflammatory cytokine release while enhancing anti-inflammatory responses. Additionally, niacin facilitates the recruitment of M2-type microglia to the periventricular region, where they promote hematoma clearance via the CD36/CD163/HO-1 axis, reducing iron deposition and oxidative stress. Importantly, niacin significantly improved both short- and long-term neurological outcomes after GMH, and this neuroprotection was dependent on microglia and at least partially mediated by the HCAR2-driven SIRT1/Nrf2 signaling cascade.

Niacin is a water-soluble vitamin and FDA-approved drug that plays a critical role in energy metabolism, particularly in maintaining NAD^+^ levels and redox homeostasis [13]. The immunomodulatory function of niacin is largely attributed to its receptor, HCAR2, which is expressed on immune cells such as macrophages and dendritic cells [30]. Previous studies have shown that HCAR2 expression in ischemic stroke models is restricted to microglia, with no expression in neurons or astrocytes [12]. Emerging evidence has demonstrated the neuroprotective effects of niacin in Parkinson's disease, Alzheimer's disease, and ischemic stroke, primarily through HCAR2 activation [12,14,31]. As a G protein–coupled receptor, HCAR2 was found in our study to be upregulated mainly in periventricular microglia after GMH, peaking at day 3, albeit with a relatively slow onset, suggesting that exogenous activation may be more effective. Its upregulation may represent an adaptive response to modulate microglial activation in association with the inflammatory and oxidative microenvironment generated by red blood cell lysis and heme degradation. To maximize CNS delivery and minimize systemic side effects, we adopted an intranasal delivery strategy to bypass the blood-brain barrier [32]. Given the short half-life of niacin, dosing was administered every 12 h [33]. No abnormal behavior was observed in any dosing group, supporting the safety of this regimen. Dose-response experiments showed that a moderate dose significantly accelerated hematoma clearance and alleviated neurological deficits by day 3, while also reducing brain edema and neuroinflammation. Long-term assessments revealed that niacin improved body weight gain and significantly enhanced cognitive and motor performance in GMH pups at 4 weeks.

Early hematoma clearance is crucial for reducing post-hemorrhagic hydrocephalus and improving GMH prognosis [21]. We found that niacin significantly reduced brain hemoglobin content and hematoma volume at days 3 and 7 after GMH, indicating a clear advantage in promoting hematoma resolution. By day 28, niacin treatment markedly alleviated ventricular dilation and reduced cortical and white matter loss, suggesting a protective effect against hydrocephalus development.

Facilitating endogenous erythrocyte clearance and enhancing microglial phagocytic capacity are important strategies for treating hemorrhagic stroke [8,21,34]. Scavenger receptors on microglia play a central role in hematoma uptake [24,26]. Our data showed that niacin upregulated the expression of CD36 and CD163 in periventricular microglia and enhanced their phagocytosis of erythrocytes and hemoglobin. Although CD163 has also been reported to mediate hemoglobin uptake in neurons, leading to iron-dependent neurotoxicity [35], our results showed colocalization of HO-1 with Hb in the periventricular region after niacin treatment. Moreover, iron staining indicated significantly reduced iron accumulation following niacin administration, suggesting that enhanced activation of the CD36/CD163/HO-1 axis promotes microglial phagocytosis and degradation of hematoma components, thereby mitigating hemoglobin breakdown–induced neurotoxicity and oxidative stress. Knockdown of HCAR2 abolished the protective effects of niacin, confirming that this receptor is required for niacin-induced neuroprotection. Furthermore, microglial depletion markedly diminished niacin-mediated hematoma resolution, neuronal preservation, and functional recovery. Primary microglia experiments also confirmed the enhancement of phagocytic activity by niacin. While microglia are key mediators of hematoma clearance, their activation may also exacerbate injury via pro-inflammatory responses. We found that niacin promoted microglial polarization toward an anti-inflammatory phenotype via HCAR2 activation, reducing the levels of pro-inflammatory cytokines in both perilesional tissue and cerebrospinal fluid.

Nrf2 is a master transcription factor regulating antioxidative defense and plays a central role in hematoma clearance after hemorrhagic stroke [28,36]. Moreover, Nrf2 facilitates microglial polarization toward the M2 phenotype and enhances their phagocytic capacity for hematoma components [8]. Our study demonstrated that Nrf2 signaling is essential for niacin-mediated neuroprotection. Niacin treatment sustained Nrf2 upregulation and facilitated its nuclear translocation, promoting microglial M2 polarization and upregulation of CD36 and CD163, thereby enhancing erythrocyte and hemoglobin phagocytosis. Concurrently, Nrf2 promoted HO-1 expression, facilitating hemoglobin degradation into non-toxic metabolites and reducing iron-induced oxidative injury. To dissect the mechanism of HCAR2-mediated neuroprotection, we examined the role of the SIRT1/Nrf2 pathway. Previous reports indicate that HCAR2 activation regulates microglial polarization through the AMPK/SIRT1 axis and suppresses NF-κB signaling [17]. In parallel, SIRT1 promotes antioxidative and anti-inflammatory responses by regulating Nrf2 [19]. To investigate the role of this pathway in our model, we administered liposome-encapsulated inhibitors via intraventricular injection. Lipo-EX527 reduced Nrf2 and its downstream targets, while Lipo-ML385 did not affect SIRT1 but effectively suppressed Nrf2 nuclear translocation and its targets. Both inhibitors impaired hematoma clearance and worsened neurological deficits, indicating that SIRT1 is a critical upstream regulator of Nrf2 activation. Although niacin replenishes NAD^+^, the expression of NAD^+^-synthesizing enzymes is reportedly low in the brain [37]. We therefore assessed whether niacin upregulates SIRT1 via HCAR2. HCAR2 knockdown suppressed the niacin-induced increase in SIRT1, and molecular docking analysis revealed high binding affinities among HCAR2, SIRT1, and Nrf2, further supporting the integrity of this signaling axis.

This study has several limitations. First, we did not assess the potential hematoma-resolving or neuroprotective roles of endogenous HCAR2 agonists, such as the short-chain fatty acid butyrate or the ketone body β-hydroxybutyrate [38]. Second, we did not assess other HO-1 degradation products, including biliverdin, bilirubin, and CO, which should be addressed in future studies to provide a more comprehensive understanding of HO-1–mediated protective mechanisms. Third, our findings are based solely on cellular and animal models, and clinical validation of the HCAR2/SIRT1/Nrf2 pathway is still needed.

## 5 Conclusion

In summary, this study demonstrates for the first time that niacin protects against GMH by activating microglial HCAR2. Through HCAR2-dependent regulation of the CD36/CD163/HO-1 axis, niacin enhances M2 microglial phagocytosis, accelerates hematoma clearance, reduces hydrocephalus, and improves neurological outcomes while mitigating neuroinflammation and oxidative stress. These effects are partly mediated via the HCAR2/SIRT1/Nrf2 pathway, highlighting niacin/HCAR2 as a promising therapeutic target for GMH.

## Data availability statement

The data supporting the findings of this study are available from the corresponding author upon reasonable request.

## CRediT authorship contribution statement

**Xiong Liu:** Investigation, Methodology, Software, Writing – original draft. **Cheng Cao:** Formal analysis, Software, Writing – original draft. **Ningbo Xu:** Data curation, Formal analysis, Writing – original draft. **Feng Xu:** Data curation, Formal analysis, Writing – original draft. **Lei Huang:** Conceptualization, Data curation, Formal analysis. **Prativa Sherchan:** Conceptualization, Data curation. **Desislava Met Doycheva:** Formal analysis, Methodology. **John H. Zhang:** Conceptualization, Project administration, Supervision, Writing – review & editing. **Jiping Tang:** Funding acquisition, Project administration, Supervision, Writing – review & editing. **Jerry J. Flores:** Investigation, Methodology, Project administration, Writing – review & editing. **Xionghui Chen:** Funding acquisition, Project administration, Supervision, Writing – review & editing.

## Declaration of competing interest

The authors declare that they have no known competing financial interests or personal relationships that could have appeared to influence the work reported in this paper.
