# Supplementary material for: Niacin enhances hematoma clearance and neurological recovery via the HCAR2/SIRT1/Nrf2 pathway after germinal matrix hemorrhage
Source: Redox Biol. 2025 Nov 4;88:103916. doi: 10.1016/j.redox.2025.103916 (PMC12793745; doi:10.1016/j.redox.2025.103916)
Supplement: Multimedia component 1 [file mmc1.docx]

**Supplementary information**

**Niacin Enhances Hematoma Clearance and Neurological Recovery via the HCAR2/SIRT1/Nrf2 Pathway After Germinal Matrix Hemorrhage**

Xiong Liu^1, 2, #^, Cheng Cao^2, 3, 4, #^, Ningbo Xu^2, 5, #^, Feng Xu^1, #^, Lei Huang^2, 6^, Prativa Sherchan^2^, Desislava Met Doycheva^2^, John H Zhang^2, 6, 7^, Jiping Tang^2, *^, Jerry J Flores^2, *^, Xionghui Chen^1, 2, *^

^1^ Department of Emergency Surgery, First Affiliated Hospital of Soochow University, Suzhou, 215000, Jiangsu, China.

^2^ Department of Physiology and Pharmacology, Basic Sciences, School of Medicine, Loma Linda University, Loma Linda, California, 92354, USA.

^3^ Department of Intensive Care Unit, The Affiliated Jiangyin Hospital of Nantong University, Jiangyin, 214400, Jiangsu, China.

^4^ Department of Brain Center, The Affiliated Jiangyin Hospital of Nantong University, Jiangyin, 214400, Jiangsu, China.

^5^ Department of Interventional Therapy, Zhujiang Hospital, Southern Medical University, Guangzhou, 510282, Guangdong, China.

^6^ Department of Neurosurgery, Loma Linda University School of Medicine, Loma Linda, California, 92354, USA.

^7^ Department of Anesthesiology and Neurology, Loma Linda University School of Medicine, Loma Linda, California, 92354, USA.

^#^ X. Liu, C. Cao, N. Xu and F. Xu, contributed equally.

^*^Corresponding Authors:

Jiping Tang, Email: [jtang@llu.edu](mailto:jtang@llu.edu)

Jerry J Flores , Email: jeflores@llu.edu

Xionghui Chen, Email: [xhchen@suda.edu.cn](mailto:xhchen@suda.edu.cn)

**Supplementary Table S1**

|  | Groups | Short-  term | Long-  term | WB Elisa | staining | HB Assay | Brain  slices | Sum |
| --- | --- | --- | --- | --- | --- | --- | --- | --- |
| EX-1 | Sham+GMH(6h,12h,24h,3d,5d,7d) |  |  | 42 |  |  |  | 422  (0% mortality rate) |
| EX-2 | Sham+GMH(3d) |  |  |  | 8 |  |  |  |
| EX-3 | Sham | 6 |  | Shared | Shared | 6 | Shared |  |
|  | GMH+Vehicle | 6 |  | Shared | Shared | 6 | Shared |  |
|  | GMH+Niacin (recommend dose/3) | 6 |  |  |  | Shared | Shared |  |
|  | GMH+Niacin (recommend dose) | 6 |  | Shared | Shared | 6 | Shared |  |
|  | GMH+Niacin (recommend dose*3) | 6 |  |  |  | Shared | Shared |  |
| EX-4 | Sham |  | 8 |  | Shared |  |  |  |
|  | GMH+Vehicle |  | 8 |  | Shared |  |  |  |
|  | GMH+Niacin (best dosage) |  | 8 |  | Shared |  |  |  |
|  | GMH+Niacin+MPN |  | 8 |  | Shared |  |  |  |
|  | GMH+Niacin+DMSO |  | 8 |  | Shared |  |  |  |
| EX-5 | Sham |  |  | 6 | 8 | 12+Shared | Shared |  |
|  | GMH+Vehicle |  |  | 6 | 8 | 12+Shared | Shared |  |
|  | GMH+Niacin (best dosage) |  |  | 6 | 8 | 12+Shared | Shared |  |
|  | GMH+Niacin+MPN |  |  | 6 | 8 | 12+Shared | Shared |  |
|  | GMH+Niacin+DMSO |  |  | 6 | 8 | 12+Shared | Shared |  |
|  | GMH+Niacin+HCAR2 CRISPR |  |  | 6 | 8 | 12+Shared | Shared |  |
|  | GMH+Niacin+Control CRISPR |  |  | 6 | 8 | 12+Shared | Shared |  |
| EX-6 | Sham+Vehicle | Shared |  |  | 4 | 6 | Shared |  |
|  | GMH+Vehicle |  |  |  | 4 |  |  |  |
|  | Sham+PLX5622 |  |  |  | 4 |  |  |  |
|  | GMH+PLX5622 |  |  |  | 4 |  |  |  |
|  | GMH+Vehicle+PLX5622 | Shared |  |  | 4 | 6 | Shared |  |
|  | GMH+Niacin+PLX5622 | Shared |  |  | 4 | 6 | Shared |  |
| EX-7 | Sham |  |  | 6 | 4 | Shared | Shared |  |
|  | GMH+Vehicle |  |  | 6 | 4 | Shared | Shared |  |
|  | GMH+Niacin (best dosage) |  |  | 6 | 4 | Shared | Shared |  |
|  | GMH+Niacin+EX527 |  |  | 6 | 4 | Shared | Shared |  |
|  | GMH+Niacin+ML385 |  |  | 6 | 4 | Shared | Shared |  |
|  | GMH+Niacin+DMSO |  |  | 6 | 4 | Shared | Shared |  |
|  | Subtotal | 30 | 40 | 120 | 112 | 120 |  |  |

Short-term: Short-term behavioral test (righting reflex/negative geotaxis)/Brain edema

Long-term: Long-term behavioral test (Morris water maze/Rotarod/foot-fault)/ weight change

Staining: immunofluorescence staining/ Iron staining/ FJC staining/ Nissl staining/ Dihydroethidium staining

WB: western blot; HB Assay: hemoglobin assay

**Supplementary Table S2**

| Drug | Drug delivery route | Dose | Company | Catalog# |
| --- | --- | --- | --- | --- |
| Niacin | i.n. | (1;3;9mg/kg) | MedChemExpress | HY-B0143 |
| Mepenzolate bromide(MPN) | i.c.v. | 5μg/pup | Sigma Aldrich | M5651 |
| HCAR2 CRISPR | i.c.v. | 2μg/pup | Santa Cruz | sc-429832 |
| Control CRISPR | i.c.v. | 2μg/pup | Santa Cruz | sc-418922 |
| PLX5622 | i.p. | 50mg/kg | MedChemExpress | HY-114153 |
| EX527 | i.c.v. | 5μg/pup | Sigma Aldrich | E7034 |
| ML385 | i.c.v. | 10μg/pup | Sigma Aldrich | SML1833 |
| Liposome | / | / | FormuMax Scientific | F10209D |
| DMSO | / | / | Sigma Aldrich | 472301 |

**Supplementary Table S3**

| Application | Antibodies | Company | Catalog# | Concentration |
| --- | --- | --- | --- | --- |
| WB | HCAR2(mouse) | Santa Cruz | sc-377292 | 1:300 |
| WB | Sirt1(mouse) | Abcam | ab110304 | 1:1000 |
| WB | Nrf2(Rabbit) | Proteintech | 16396-1-AP | 1:6000 |
| WB | CD68(Rabbit) | Abcam | ab125212 | 1:1000 |
| WB | CD206(Rabbit) | Abcam | ab64693 | 1:1000 |
| WB | CD163(Rabbit) | Abcam | ab182422 | 1:1000 |
| WB | CD36(mouse) | Santa Cruz | sc-7309 | 1:500 |
| WB | HO-1(Rabbit) | Abcam | ab13243 | 1:1000 |
| WB | MPO(Rabbit) | Abcam | ab208670 | 1:1000 |
| WB | IL-1β(Rabbit) | Abcam | ab254360 | 1:500 |
| WB | TNF-α(mouse) | Proteintech | 60291-1-Ig | 1:2000 |
| WB | IL-6(mouse) | Abcam | ab9324 | 1:500 |
| WB | IL-10(Rabbit) | Abcam | ab9969 | 1:500 |
| WB | Lamin B1(Rabbit) | Abcam | ab16048 | 1:1000 |
| WB | GAPDH | Proteintech | 60004-1-Ig | 1:50000 |
| WB | β-Actin(mouse) | Santa Cruz | sc-47778 | 1:1000 |
| WB | mouse anti-rabbit  IgG-HRP | Santa Cruz | sc-2357 | 1:2000 |
| WB | m-IgG Fc BP-HRP | Santa Cruz | sc-525409 | 1:2000 |
| IF | Iba1(mouse) | Abcam | ab283319 | 1:200 |
| IF | NeuN(mouse) | Abcam | ab104224 | 1:200 |
| IF | GFAP(goat) | Abcam | ab53554 | 1:200 |
| IF | HCAR2(Rabbit) | Thermo Fisher | PA5-90579 | 1:50 |
| IF | Nrf2(Rabbit) | Proteintech | 16396-1-AP | 1:100 |
| IF | CD163 | Abcam | ab182422 | 1:100 |
| IF | CD36 | Abcam | ab252922 | 1:100 |
| IF | HO-1(Rabbit) | Abcam | ab13243 | 1:100 |
| IF | HO-1(mouse) | Abcam | ab13248 | 1:100 |
| IF | Hemoglobin(Rabbit) | Abcam | ab92492 | 1:100 |
| IF | IL-1β(Rabbit) | Abcam | ab254360 | 1:100 |
| IF | TNF-α(Rabbit) | Abcam | ab205587 | 1:100 |
| IF | Secondary Antibodies | Jackson ImmunoResearch | 711-095-152  715-295-150  705-295-003  155-095-044  111-295-003 | 1:400 |

WB: western blot; IF:immunofluorescence


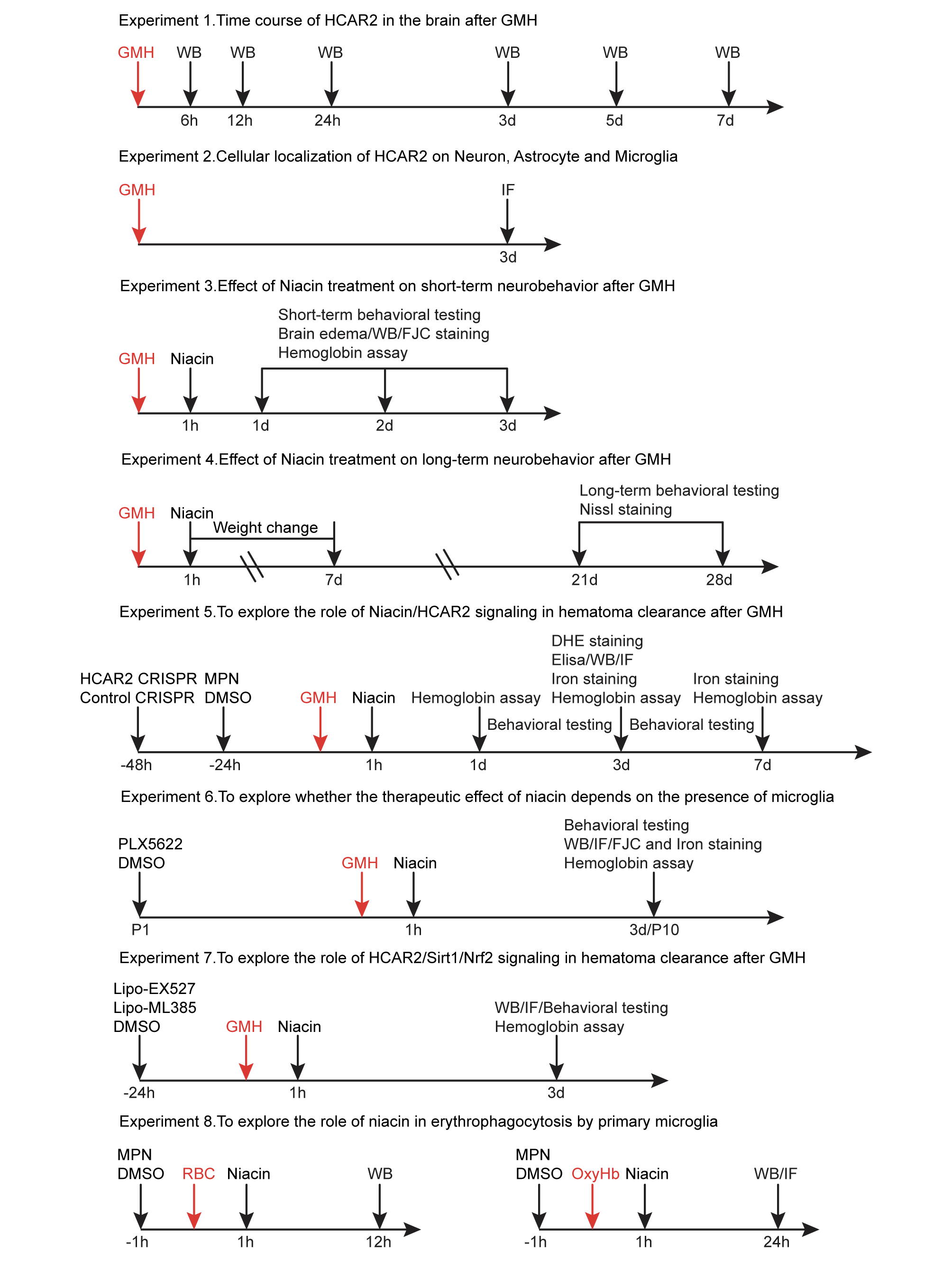


**Supplemental figure S1. Timeline of experiment design.**


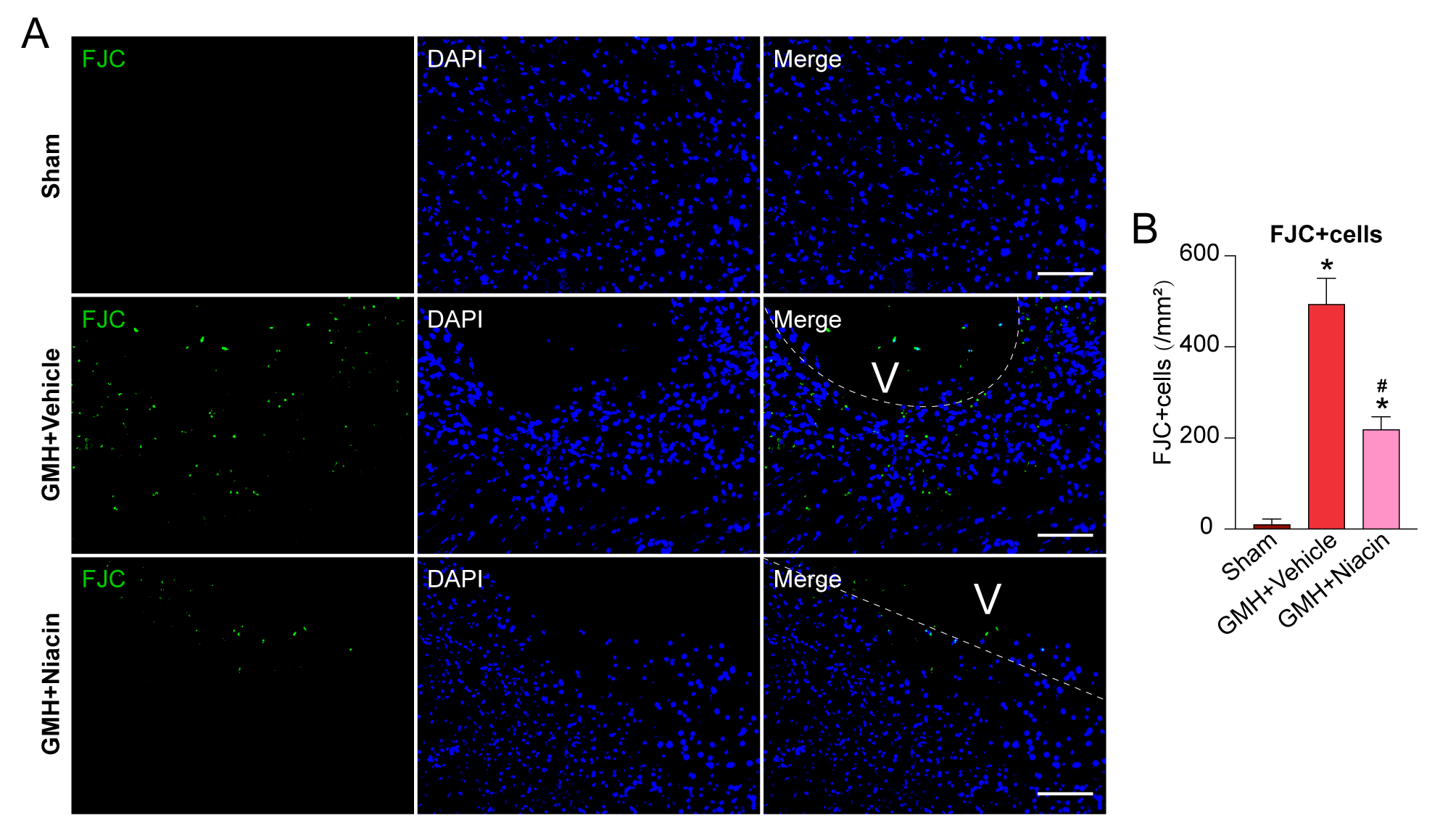


**Supplemental figure S2. A-B**, Representative images of FJC staining and quantitative analysis. Scale bar = 100 µm. n = 4 per group. Values are expressed as mean ± SD. One-way ANOVA followed by Tukey’s test. n=4 per group. *P<0.05 vs Sham, #P<0.05 vs GMH + Vehicle. V, Ventricles.


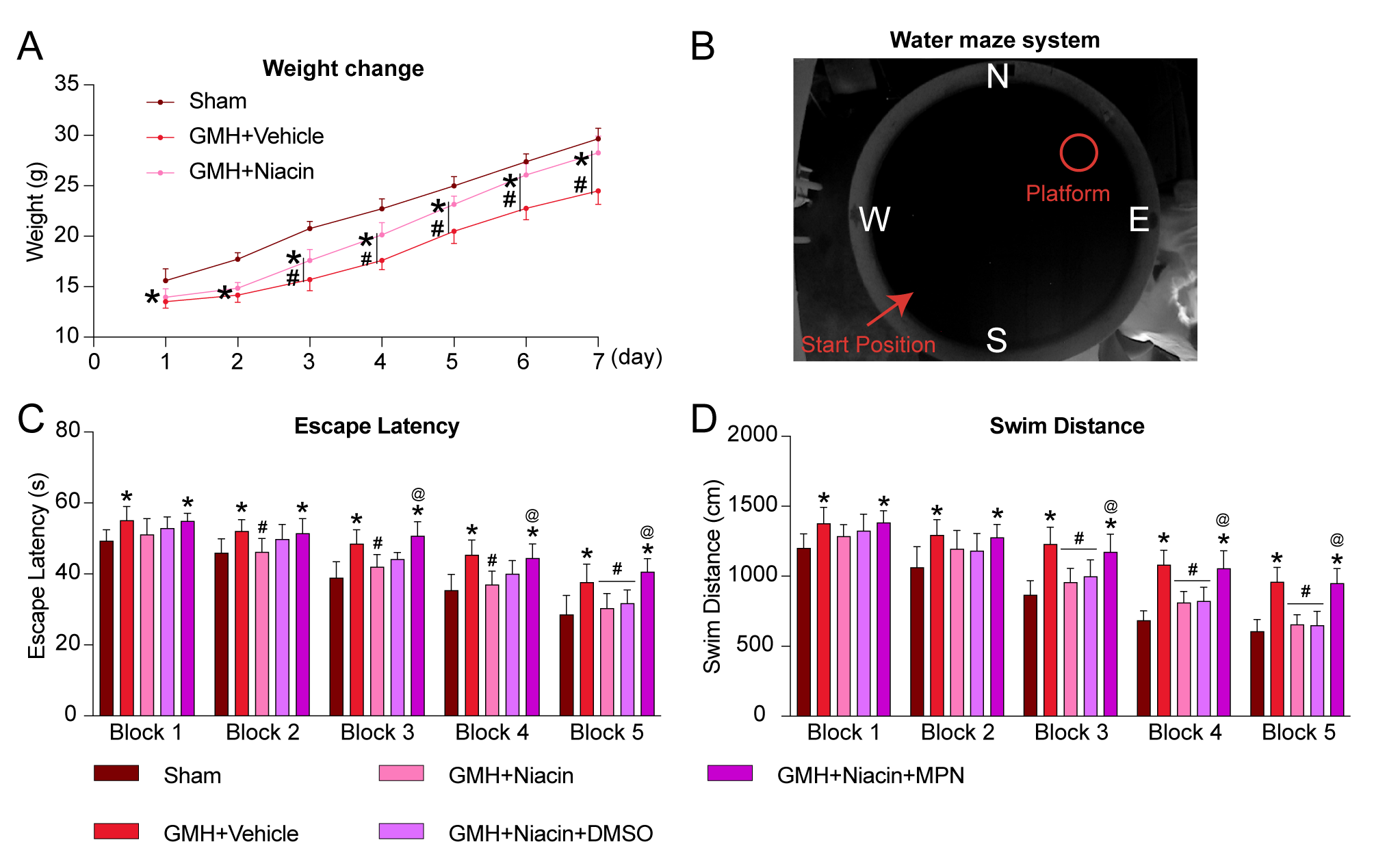


**Supplemental figure S3. A**, Body weight changes in pups from day 1 to day 7 after GMH. n = 8 per group. **B**, Water maze system. **C-D**, Escape latency and swimming distance on days 22–26 after GMH. Values are expressed as mean ± SD. One-way ANOVA followed by Tukey’s test. n=8 per group. *P<0.05 vs Sham, #P<0.05 vs GMH + Vehicle, @P < 0.05 vs. GMH + Niacin or GMH + Niacin + dimethyl sulfoxide (DMSO). MPN, Mepenzolate bromide.


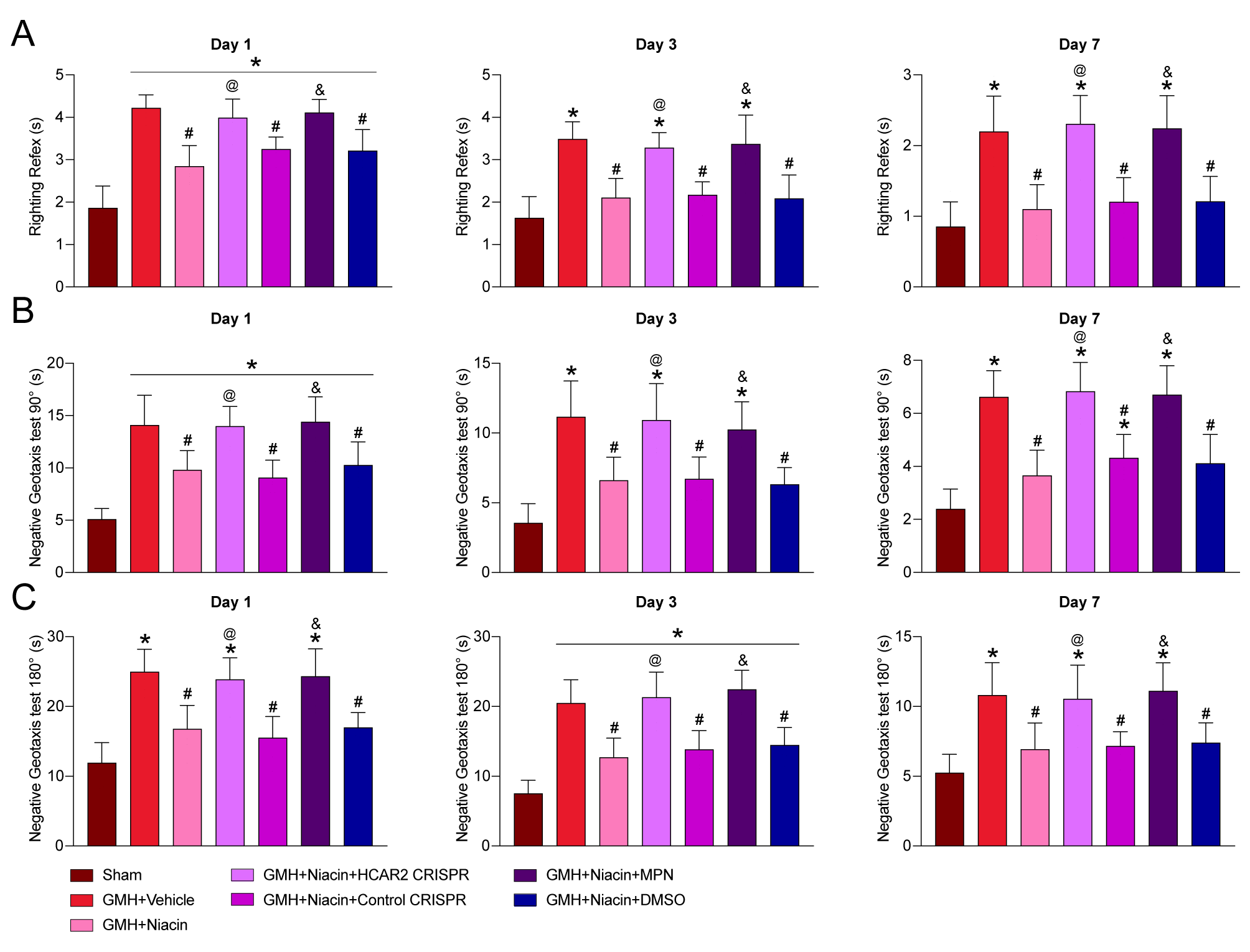


**Supplemental figure S4. A-C**, The effects of HCAR2 activation and knockout on short-term neurological function in GMH pups. Values are expressed as mean ± SD. One-way ANOVA followed by Tukey’s test. n=6 per group. *P < 0.05 vs. Sham; #P < 0.05 vs. GMH + Vehicle; @P < 0.05 vs. GMH + Niacin or GMH + Niacin + Control CRISPR; &P < 0.05 vs. GMH + Niacin or GMH + Niacin + dimethyl sulfoxide (DMSO). MPN, Mepenzolate bromide.


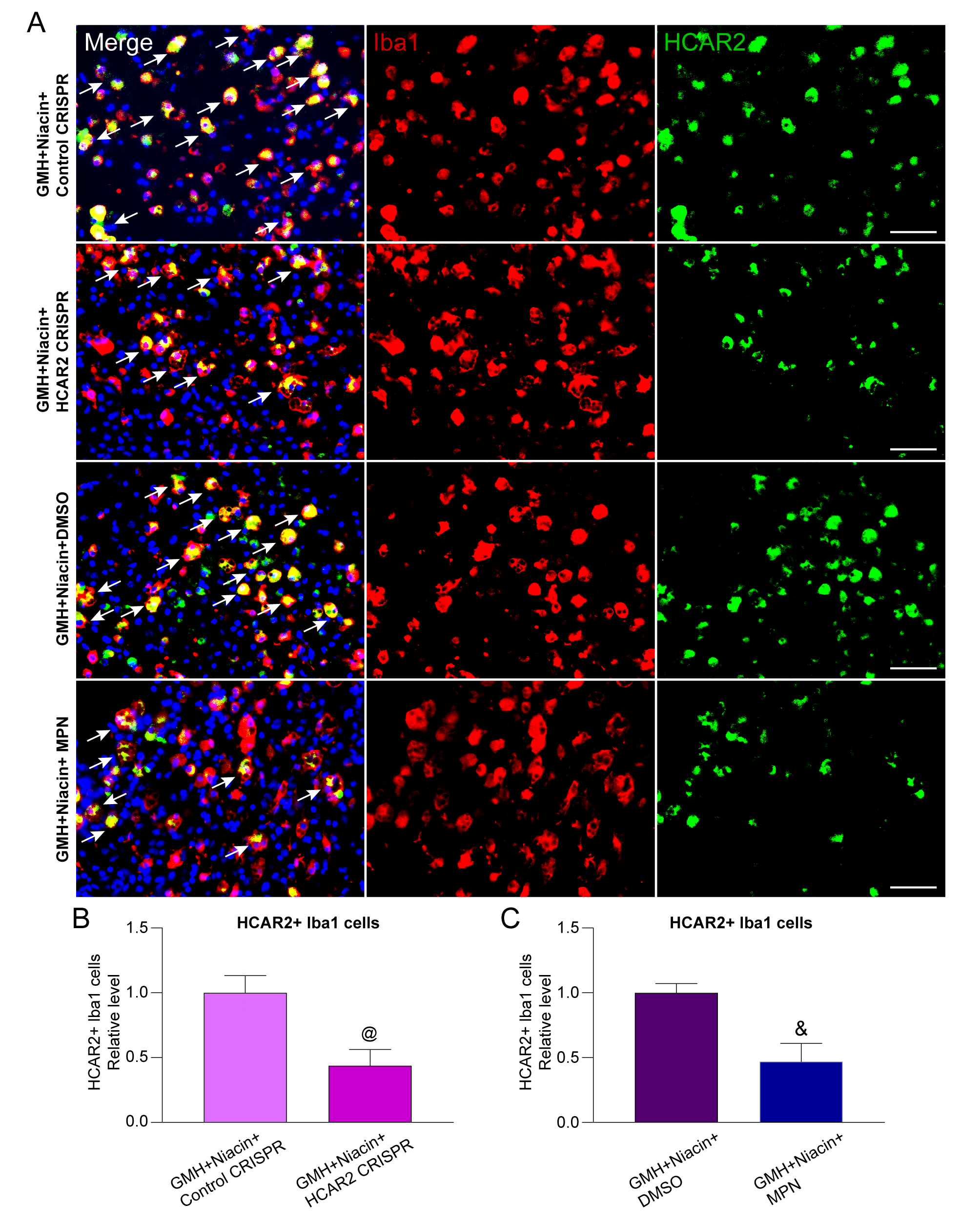


**Supplemental figure S5. A.** Representative immunofluorescence images showing HCAR2-positive microglia (HCAR2⁺, green; Iba1⁺ [ionized calcium-binding adaptor molecule 1], red) in the periventricular region after GMH. Scale bar = 50 µm. n = 4 per group. **B-C**, Quantitative analysis of HCAR2⁺ microglia. Values are expressed as mean ± SD. n = 4 per group. @*P* < 0.05 vs. GMH + Niacin + Control CRISPR. &*P* < 0.05 vs. GMH + Niacin + dimethyl sulfoxide (DMSO). MPN, Mepenzolate bromide.


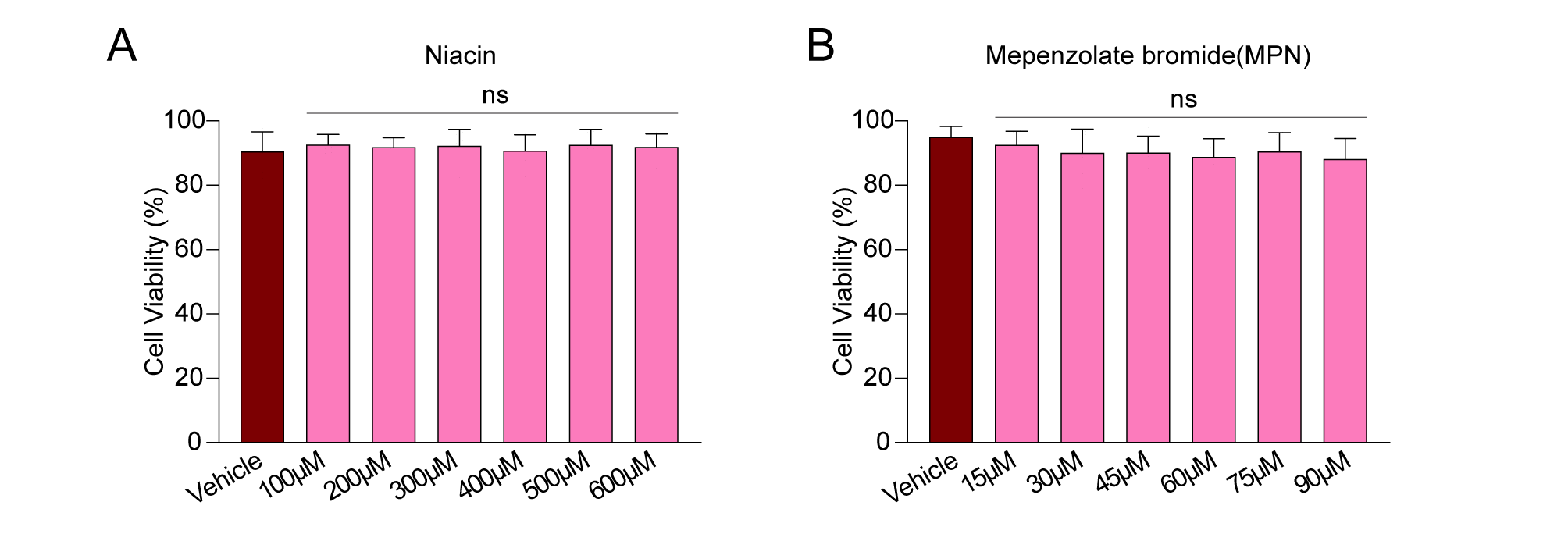


**Supplemental figure S6. A-B**, Cell viability. Values are expressed as mean ± SD. n = 6 per group. *P < 0.05 vs. Vehicle.


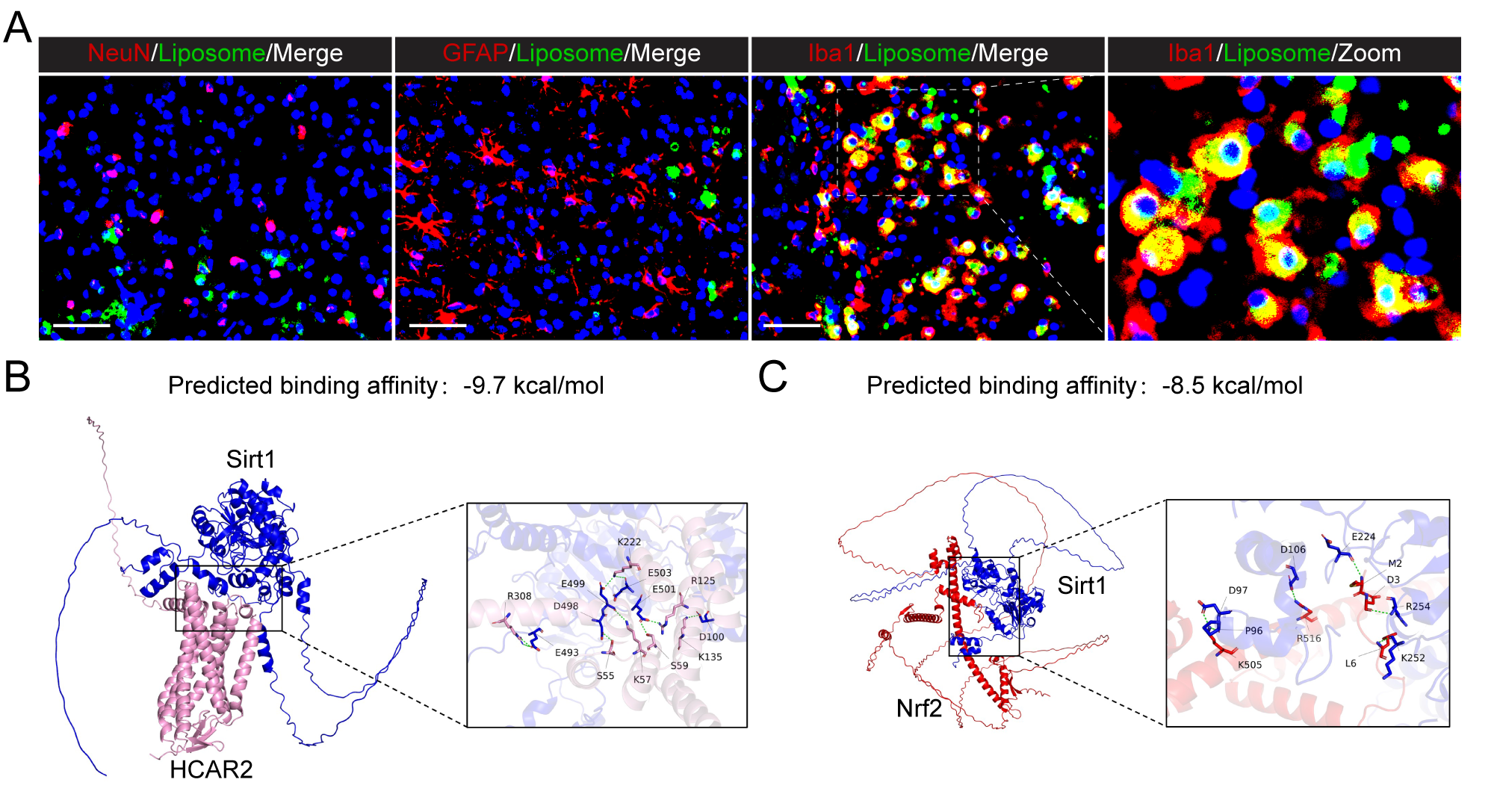


**Supplemental figure S7. A**, Representative immunofluorescence images showing successful delivery of the liposome-encapsulated inhibitor to microglia in the cerebral ventricles of neonatal pups. **B-C**, Molecular docking results.

**
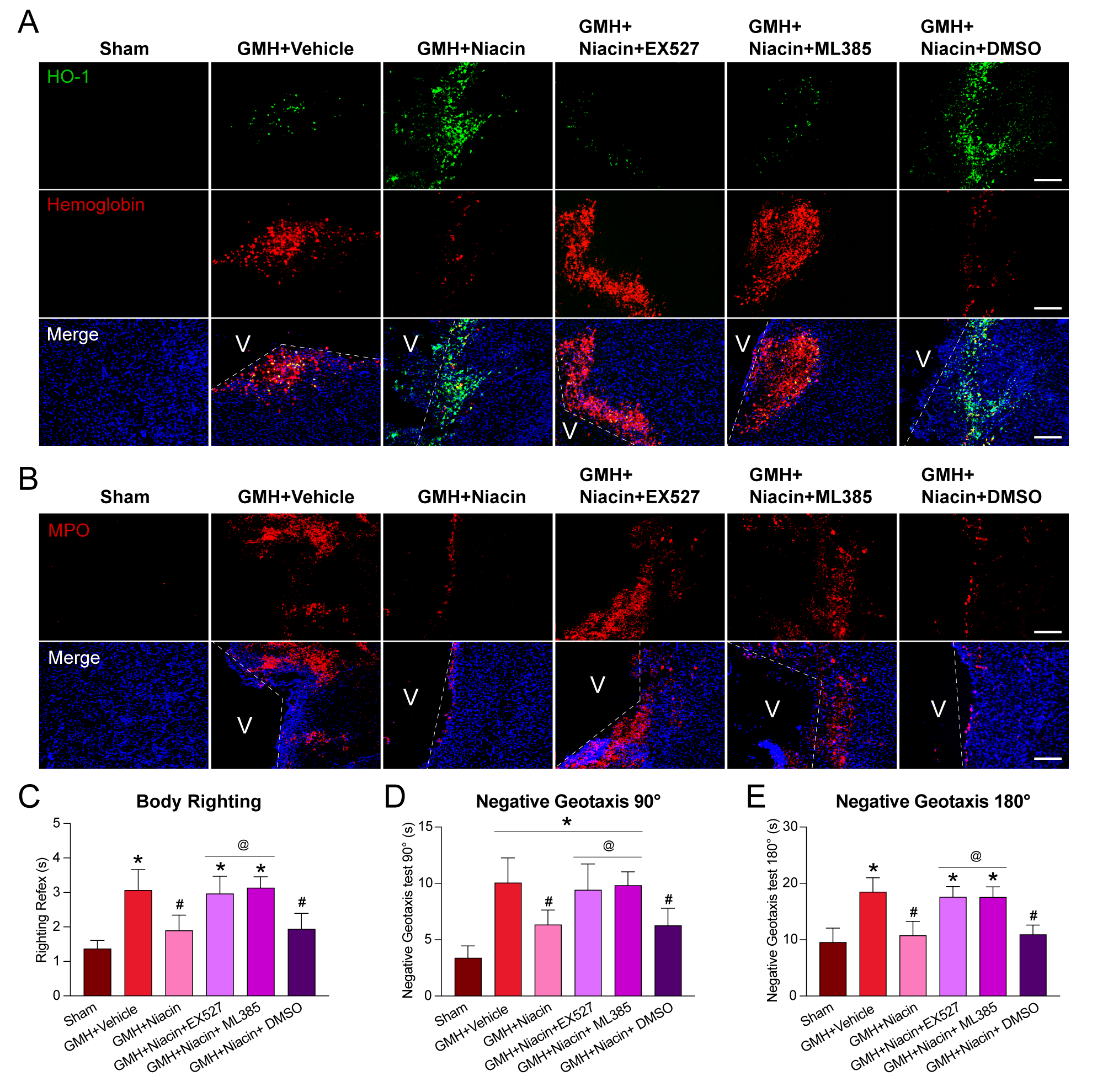
**

**Supplemental figure S8. A**, Representative immunofluorescence images showing colocalization of HO-1 with hemoglobin in the periventricular region after GMH. Scale bar = 200 µm. n = 4 per group. **B**, Representative immunofluorescence images showing MPO⁺ neutrophils in the ventricular region. Scale bar = 200 µm. n = 4 per group. **C-E**, Inhibition of the Sirt1/Nrf2 signaling pathway aggravated short-term neurological deficits in neonatal pups. Values are expressed as mean ± SD. One-way ANOVA followed by Tukey’s test. n=6 per group. *P < 0.05 vs. Sham; #P < 0.05 vs. GMH + Vehicle; @P < 0.05 vs. GMH + Niacin or GMH + Niacin + dimethyl sulfoxide (DMSO). V, Ventricles.

**
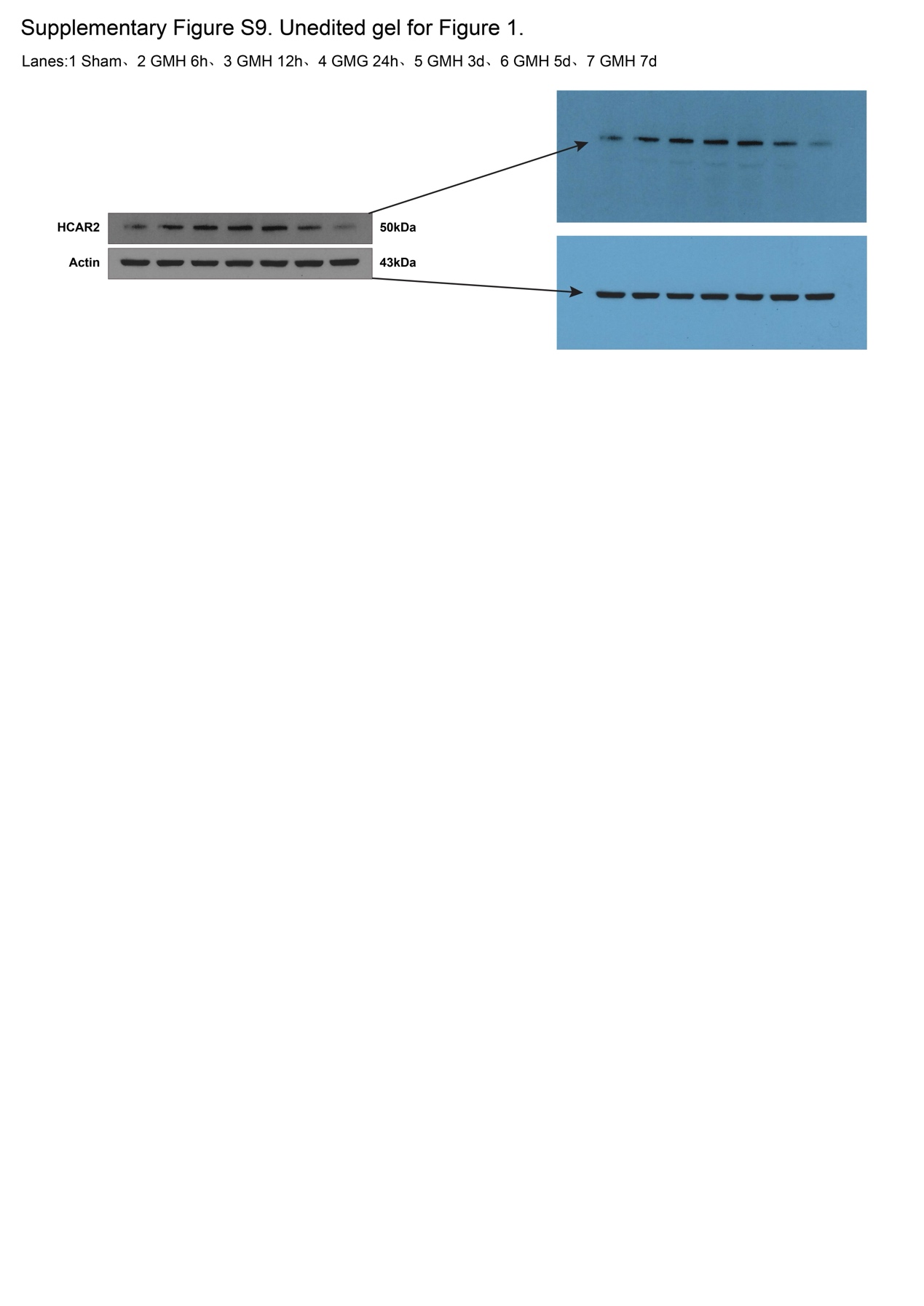
**

**
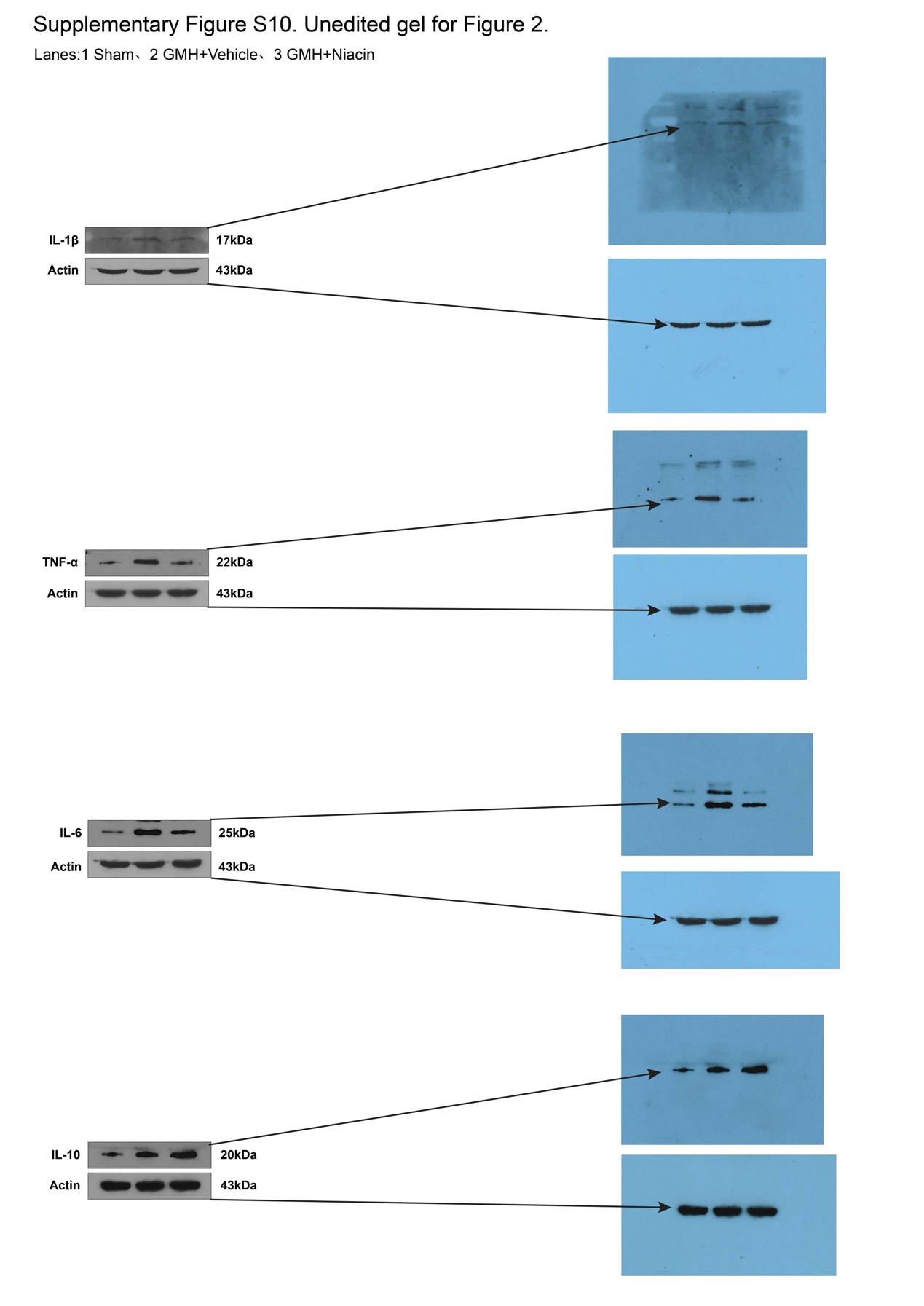
**

**
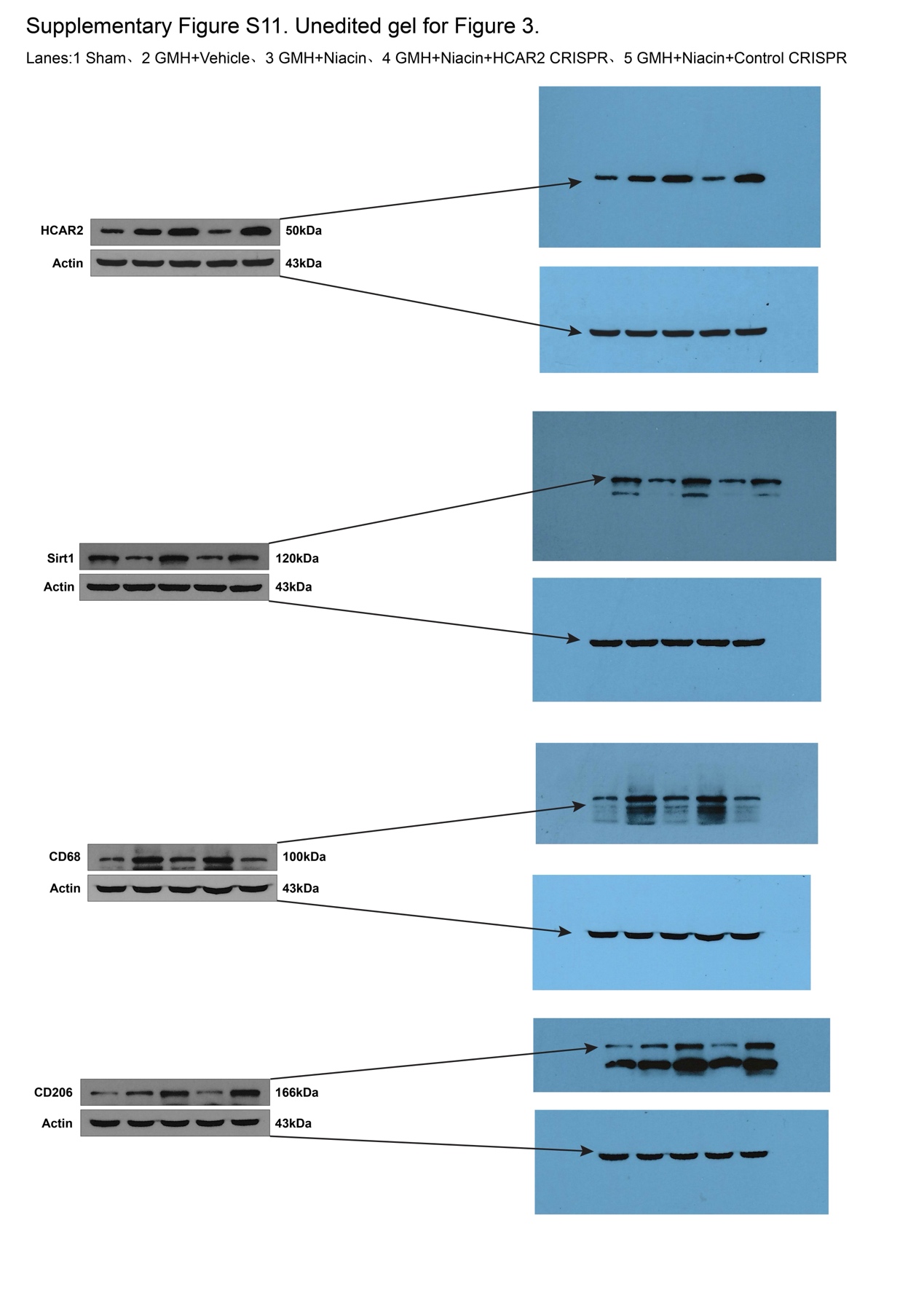
**

**
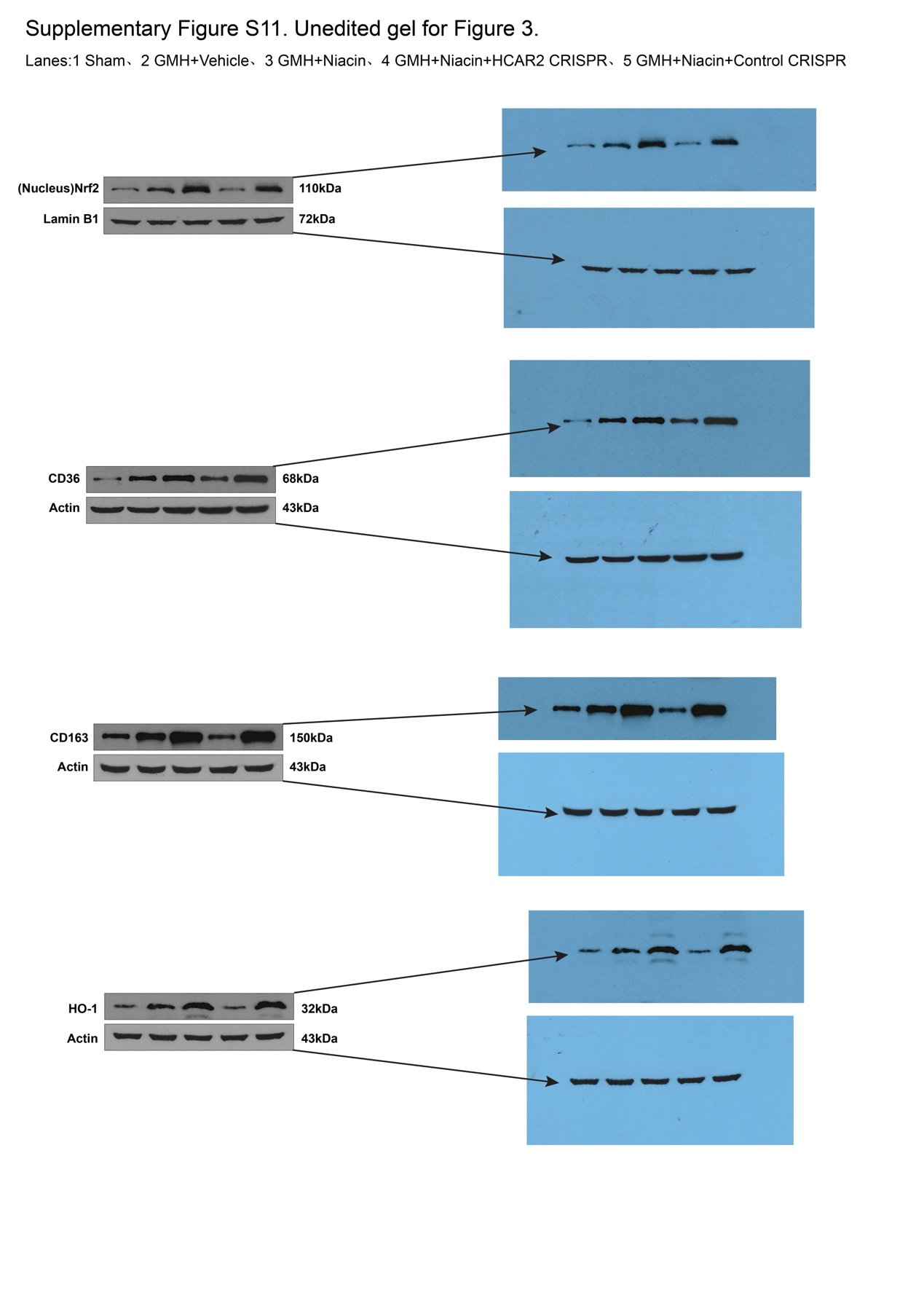
**

**
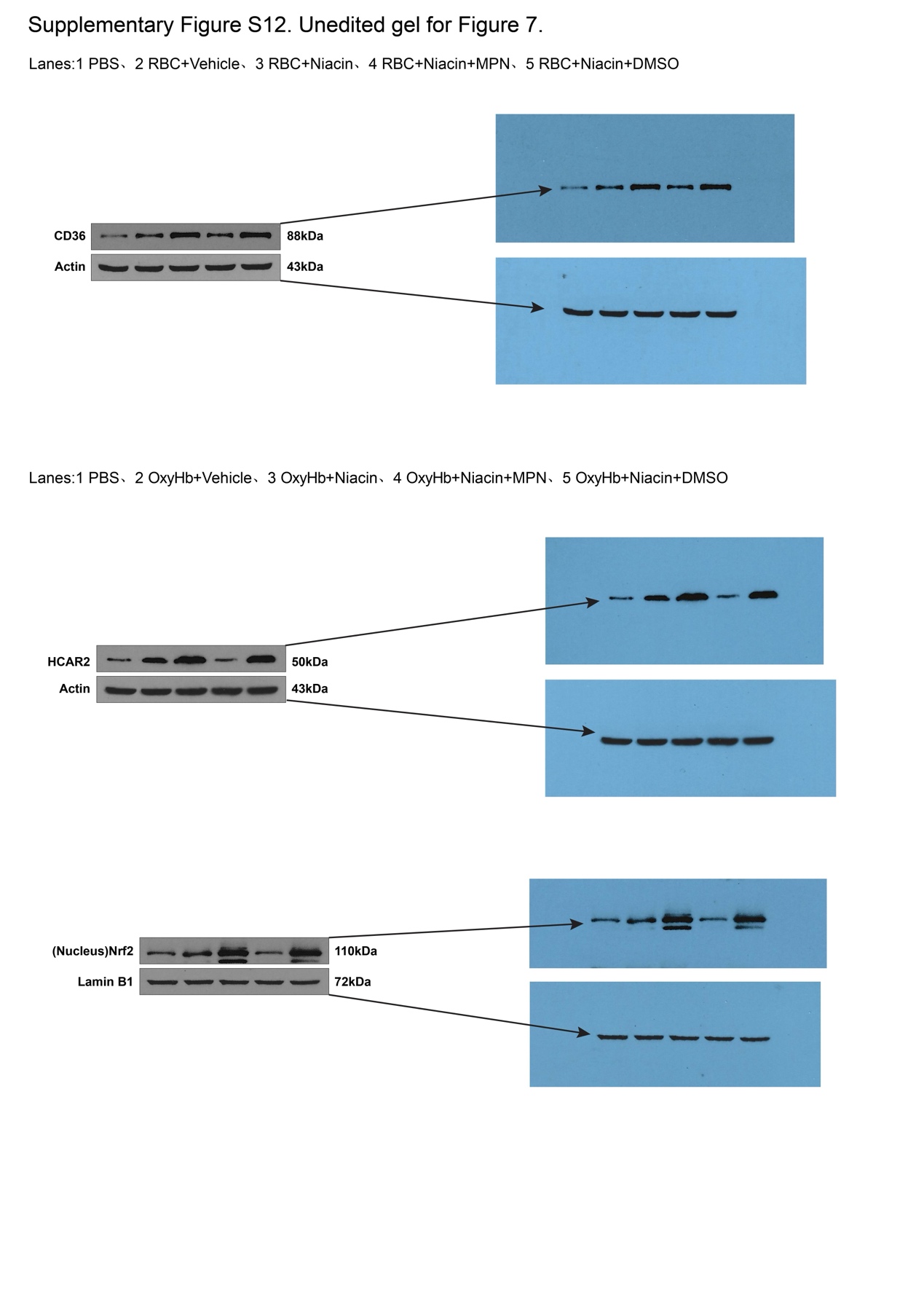
**

**
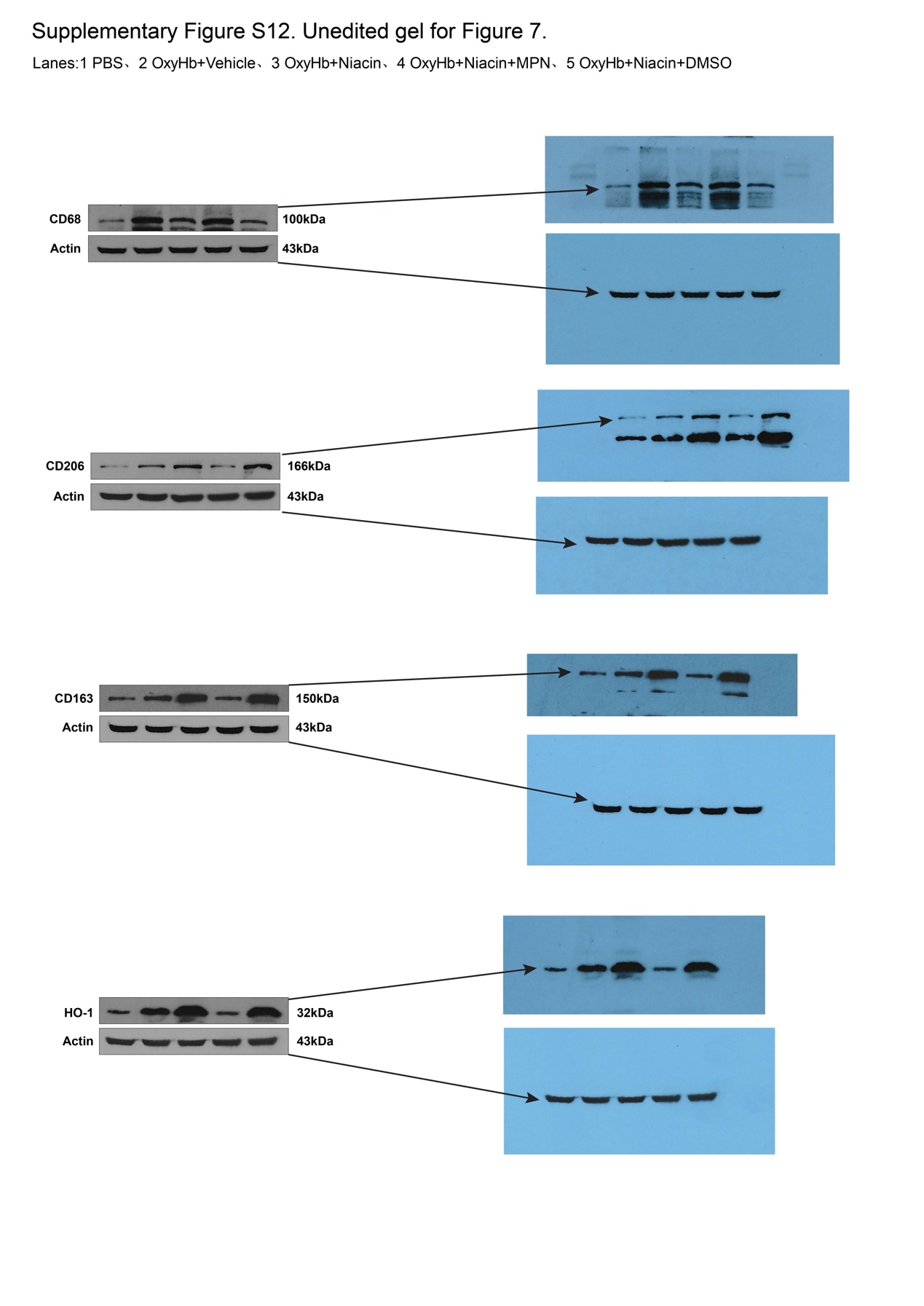
**

**
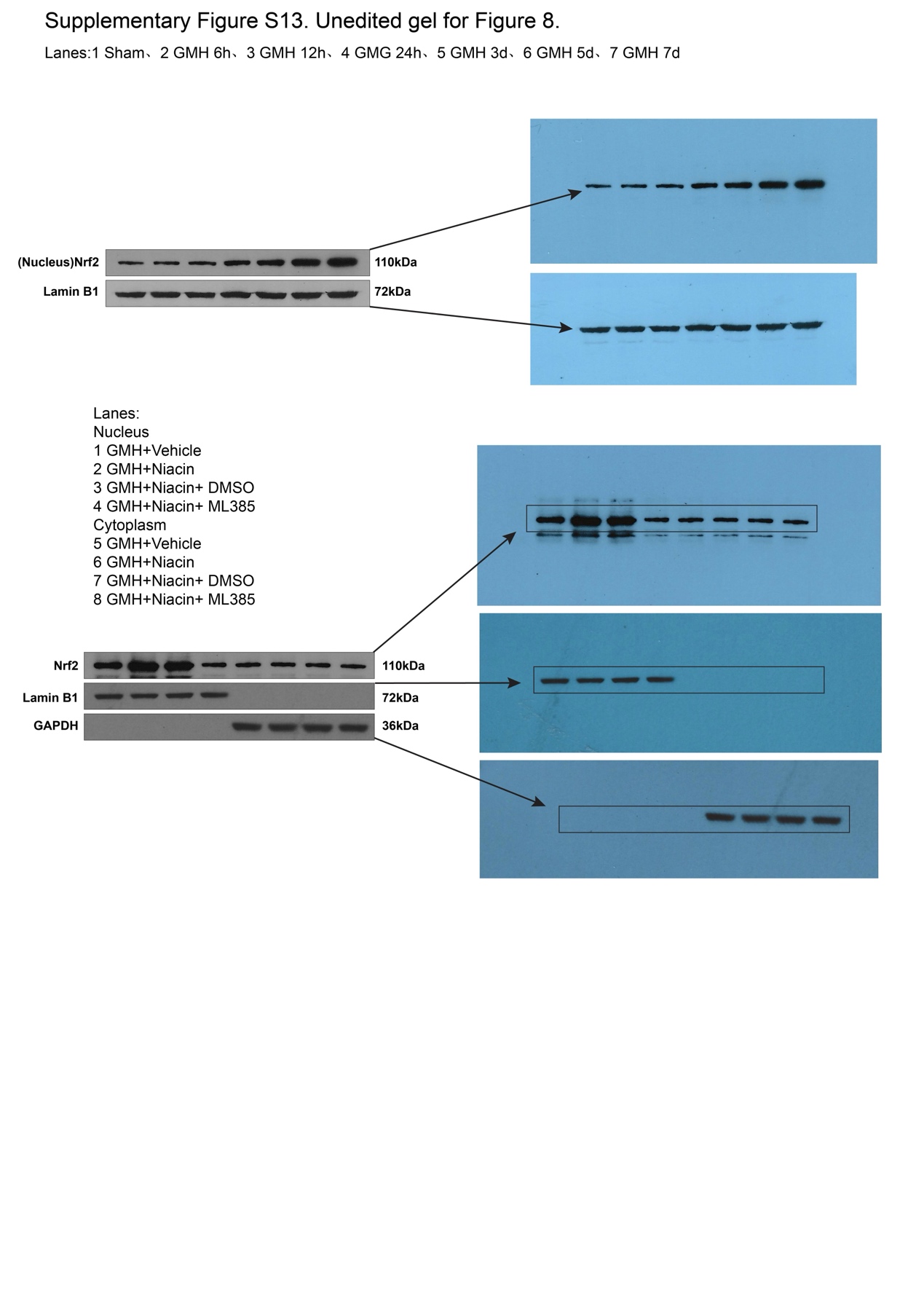
**

**
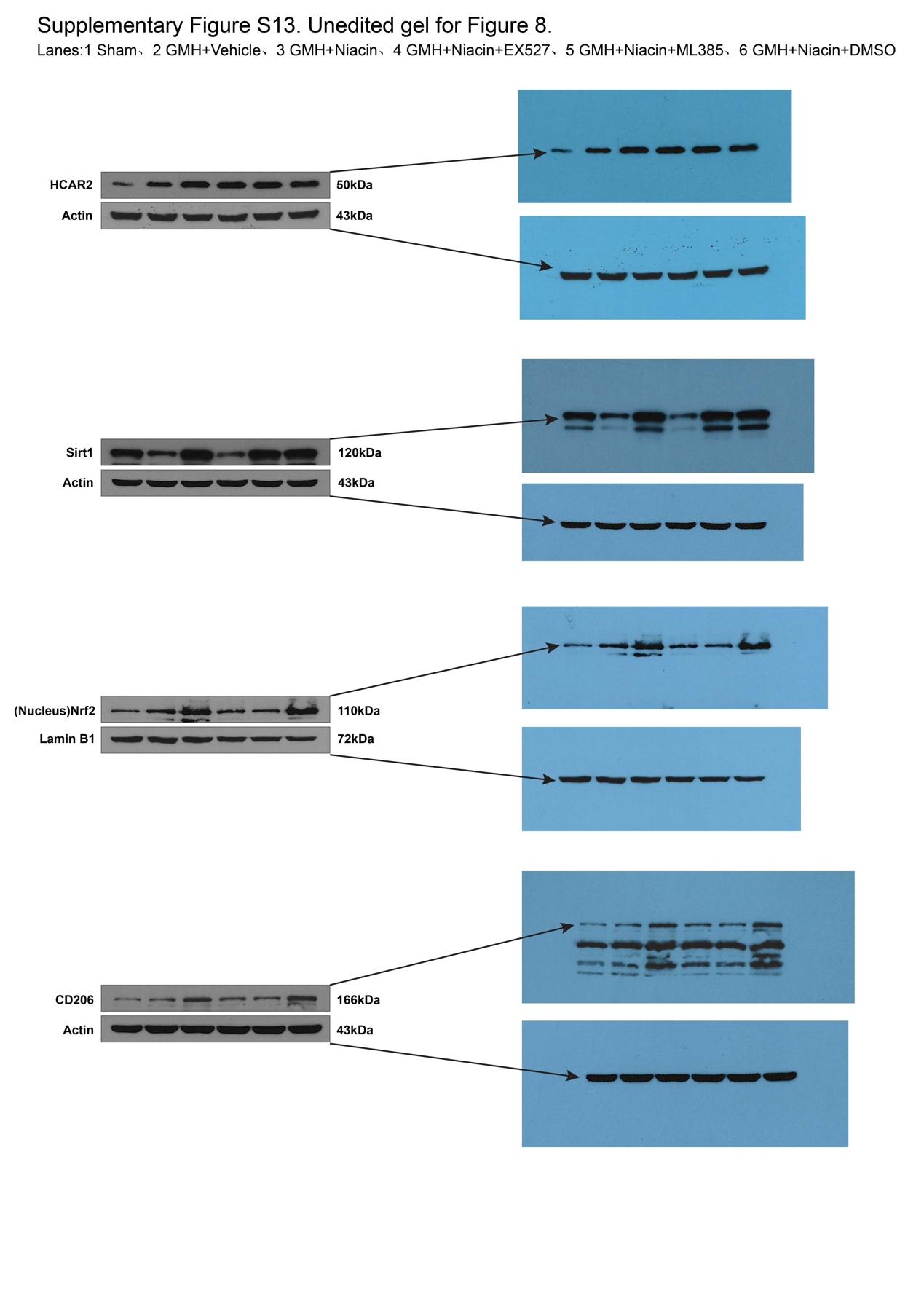
**

**
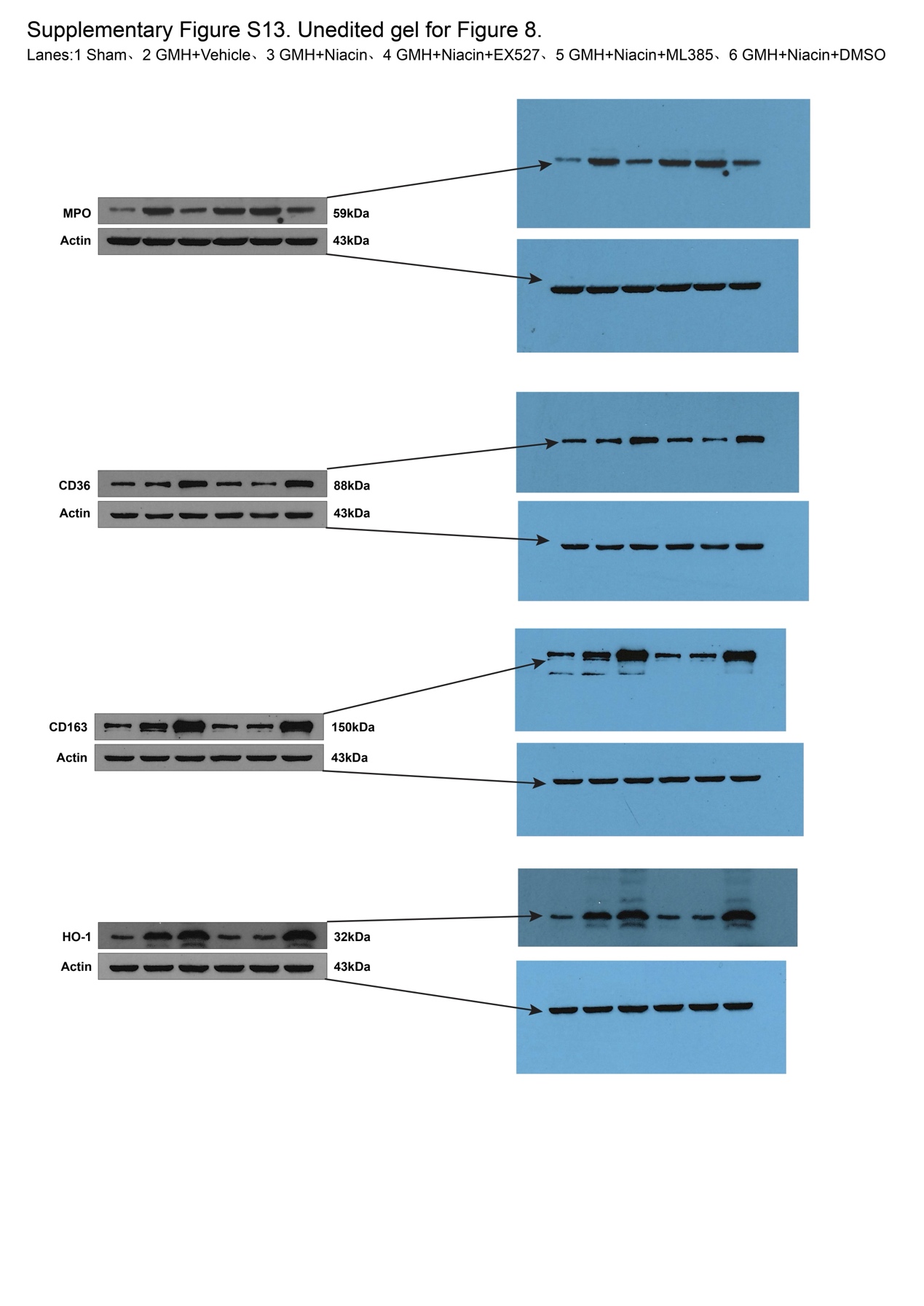
**
